# Addition of aluminium, zinc and magnesium hydrides to rhodium(iii)†
†Electronic supplementary information (ESI) available. CCDC 1047853–1047855 1056989. For ESI and crystallographic data in CIF or other electronic format see DOI: 10.1039/c5sc01309g



**DOI:** 10.1039/c5sc01309g

**Published:** 2015-07-03

**Authors:** Olga Ekkert, Andrew J. P. White, Harold Toms, Mark R. Crimmin

**Affiliations:** a Department , of Chemistry , Imperial College London , South Kensington , London , SW7 2AZ , UK . Email: m.crimmin@imperial.ac.uk; b The School of Biological and Chemical Sciences , Queen Mary, University of London , Mile End Road , London E1 4NS , UK

## Abstract

We report the addition of M–H bonds (M = Al, Zn, Mg) to a Rh(iii) intermediate generated from the reductive elimination of triethylsilane from [Cp*Rh(H)_2_(SiEt_3_)_2_]. A series of new heterobimetallic complexes possessing Rh–M bonds have been isolated and characterised by a number of spectroscopic (^1^H, ^29^Si, ^13^C, ^103^Rh NMR, infrared, and X-ray diffraction) and computational techniques (NBO and QTAIM analysis). Experimental and computational data are consistent with cleavage of the M–H bond upon addition to rhodium with formation of new Rh–M and Rh–H bonds. Upon photolysis the Al analogue of this series undergoes a further elimination reaction producing triethylsilane and a highly unusual Rh_2_Al_2_H_4_ containing cluster proposed to contain an Al(i) bridging ligand.

## Abstract 2

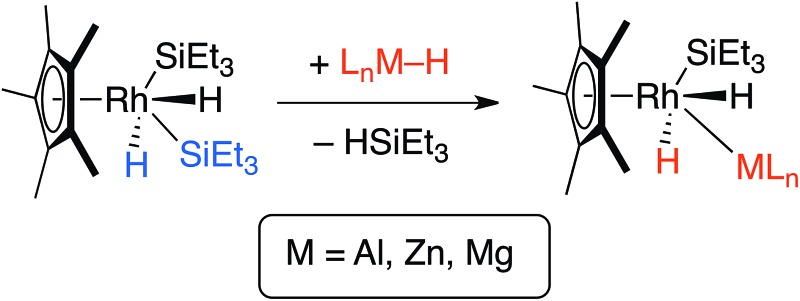
We report the addition of M–H bonds (M = Al, Zn, Mg) to a Rh(iii) intermediate generated from the reductive elimination of triethylsilane from [Cp*Rh(H)_2_(SiEt_3_)_2_].

## Introduction

This paper concerns the addition of main group hydrides to a transition metal complex. We have studied the addition of M–H (M = Al, Zn, Mg) bonds to a 16-electron Rh(iii) fragment and compared our data to literature in which B–H or Si–H bonds add to the same species. We demonstrate that in combination the transformations may be defined by a spectrum of reactivity that lies between two extreme definitions: oxidative addition and hydride transfer. We show that as electronegativity difference between the M and H atoms increases, the description of hydride transfer becomes more accurate than oxidative addition.

The oxidative addition of element–hydrogen bonds to transition metal centres is a reaction of fundamental importance to catalysis. Upon coordination of H_2_ to a transition metal, back-bonding can result in a lengthening of the H–H bond and the formation of stretched dihydrogen adducts *in lieu* of traditional dihydrogen complexes.^1,2^ In the extreme, cleavage of the H–H bond and oxidative addition to the metal centre can occur.^3^ While the coordination and oxidative addition of H–H, Si–H and B–H bonds to transition metals continues to receive considerable attention,^3–5^ only recently have heavier main group hydrides begun to emerge as ligands for transition metal complexes.^6^ For example, the coordination of Al–H bonds to group 5, 6, 10 and 11 metals has now been reported.^7–9^ In the majority of cases, these species represent classical σ-complexes with donation of the electron-pair to the transition metal and retention of a significant Al–H bond. In a single case, Aldridge and co-workers have shown that stretching of Al–H bonds may occur upon coordination to a cobalt carbonyl fragment derived from [Co_2_(CO)_8_].^8*d*^ Despite these findings, little is known about the activation of heavier main group hydrides at transition metal centres, and less still about the heterobimetallic complexes that would result from hydride transfer from the main group to the transition metal. Herein we report the addition of M–H bonds (M = Al, Zn, Mg) to a Rh(iii) intermediate generated from the reductive elimination of triethylsilane from [Cp*Rh(H)_2_(SiEt_3_)_2_].

## Results and discussion

**Scheme 1:**
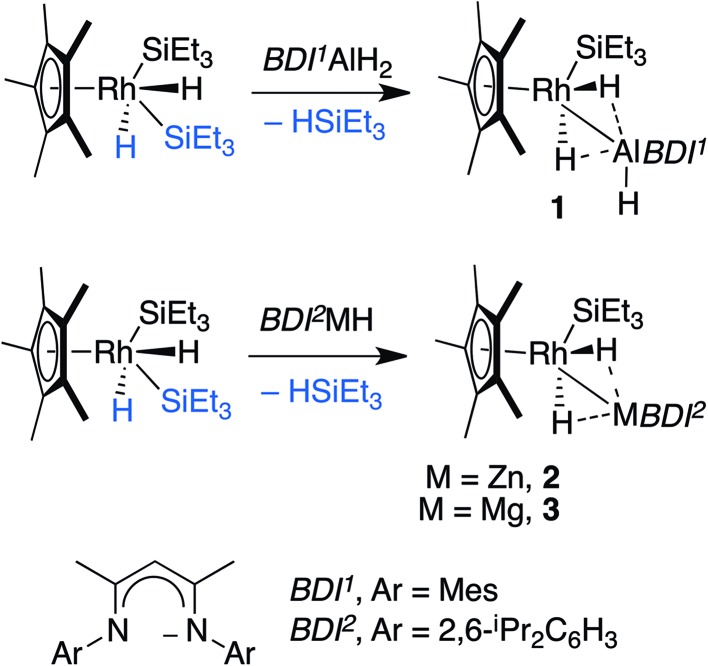
Reaction of Al, Zn and Mg hydrides with [Cp*Rh(H)_2_(SiEt_3_)_2_].

Hartwig and co-workers have shown that H–Bpin reacts with [Cp*Rh(H)_2_(SiEt_3_)_2_] under thermal or photochemical conditions to give the corresponding metal boryl complex by B–H activation.^10^ In line with these findings, thermolysis of [Cp*Rh(H)_2_(SiEt_3_)_2_] in the presence of β-diketiminate stabilised aluminium, magnesium or zinc hydrides in C_6_D_6_ solution led to the formation of the corresponding heterobimetallic complexes **1–3** along with a single equiv. of triethylsilane (Scheme 1).^11,12^

Complexes **1–3** were isolated following crystallisation from *n*-hexane or hexamethyldisiloxane at –35 °C and have been characterised by multinuclear NMR and infrared spectroscopy, CHN analysis, and single crystal X-ray diffraction.^13^ ^1^H NMR experiments in toluene-d_8_ revealed that **1** demonstrates a broad doublet at *δ* = –15.14 ppm (^1^ *J*_Rh–H_ = 40.2 ppm Hz, fwhm = 13.2 Hz), while complexes **2** and **3** show sharp signals at *δ* = –14.28 (^1^ *J*_Rh–H_ = 34.6 Hz, fwhm = 4.9 Hz) and –15.91 ppm (^1^ *J*_Rh–H_ = 40.5 Hz, fwhm = 4.9 Hz) respectively. For comparison the hydride resonances of the starting material *BDI* ^*1*^AlH_2_ are observed at *δ* = 4.62 ppm (fwhm = 170 Hz). The chemical shift, significant Rh–H coupling and small line broadening by ^27^Al (100%, I = 5/2) in **1** suggests that the hydrides are located on rhodium with only a weak interaction with the main group element (*vide infra*). The terminal aluminium hydride resonance of **1** was located by VT NMR experiments (see ESI†).

Maitlis and co-workers have characterised [Cp*Rh(H)_2_(SiEt_3_)_2_] by neutron diffraction experiments and assigned it as a Rh(v) complex.^11a–b^ While Hartree–Fock calculations support this formulation,^11*c*^ subsequent calculations suggest that assignment of the +5 formal oxidation state may be misleading due to the fact that the Si···H distance may be compressed with very little energetic cost.^11*d*^ ^103^Rh–^1^H HMBC experiments show that this latter species (*δ* = –1709 ppm) has a similar magnetic environment at rhodium to the heterobimetallic complexes we have isolated (**1**, *δ* = –1570 ppm; **2**, *δ* = –1743 ppm; **3**, *δ* = –1540 ppm). The absence of H···H bonding was supported by long *T*_1_ relaxation times of the Rh–H resonances of **1–3** (*T*_1_ = 0.9–1.0 s). The lack of a strong M–H interaction was further evidenced by infrared data on **1–3**. The Rh–H stretches (**1**, 1966 cm^–1^; **2**, 1959 cm^–1^; **3**, 1929 cm^–1^) are shifted to slightly lower energy relative to that in [Cp*Rh(H)_2_(SiEt_3_)_2_] (2019 cm^–1^).^11^

**Fig. 1:**
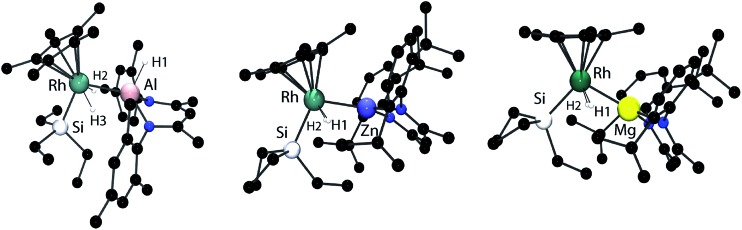
The crystal structures of **1** (left), **2** (middle) and **3** (right). Selected bond angles and bond lengths. 1: Rh–Si 2.3402(8), Rh–Al 2.4527(8), Si–Rh–Al 102.82(3). 2: RhSi 2.3571(8), Rh–Zn 2.4158(4), Si–Rh–Zn 106.86(2). 3: Rh–Si 2.3437(7), Rh–Mg 2.5049(8), Si–Rh–Mg 106.23(2). Hydrides were located within the Fourier difference maps.

Single crystal X-ray diffraction of samples of **1–3** confirmed the assigned structures. The Rh–Al, Rh–Zn and Rh–Mg distances are all within the sum of the covalent radii, taking values of 2.4527(8), 2.4158(4) and 2.5049(8) Å respectively.^14^ For comparison, whether hydride bridged or not, Rh–Zn distances in multimetallic clusters range from 2.45–2.58 Å and are longer than those found in **2**.^15,16^ Similarly the Rh–Al bond length of **1** is shorter than the Rh→Al interaction in [Cp*Rh(PMe_3_)_2_(Al_2_Me_2_Cl_2_)], and is similar to that found in a Rh/Al heterobimetallic recently isolated by our group.^17,18^ To the best of our knowledge, no examples of crystallographically characterised complexes containing a Mg–Rh bond exist. While the element–hydrogen bond lengths should be treated with caution it is noteworthy that in all cases the Rh–H bond lengths are short (1.42(3)–1.56(3) Å) and the M–H distances are long (2.06(3)–2.17(3) Å). For comparison the Al–H bond length of the terminal hydride in **1** is 1.65(2) Å (Fig. 1).

**Fig. 2:**
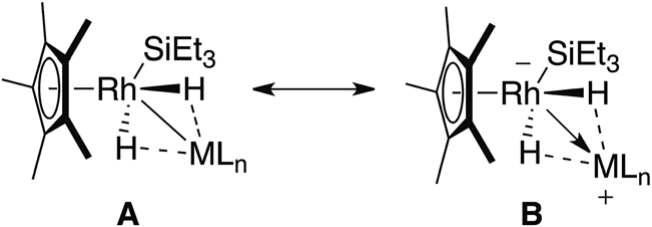
Simplified extreme bonding descriptions in **1–3**, [Cp*Rh(H)_2_(SiEt_3_)_2_] and [Cp*Rh(H)_2_(SiEt_3_) (Bpin)]. Dotted lines represent the weak interaction between H and M, these could also be represented by a half-arrow from the hydride to M.^6^ M = B, Si, Al, Zn, Mg.

**Fig. 3:**
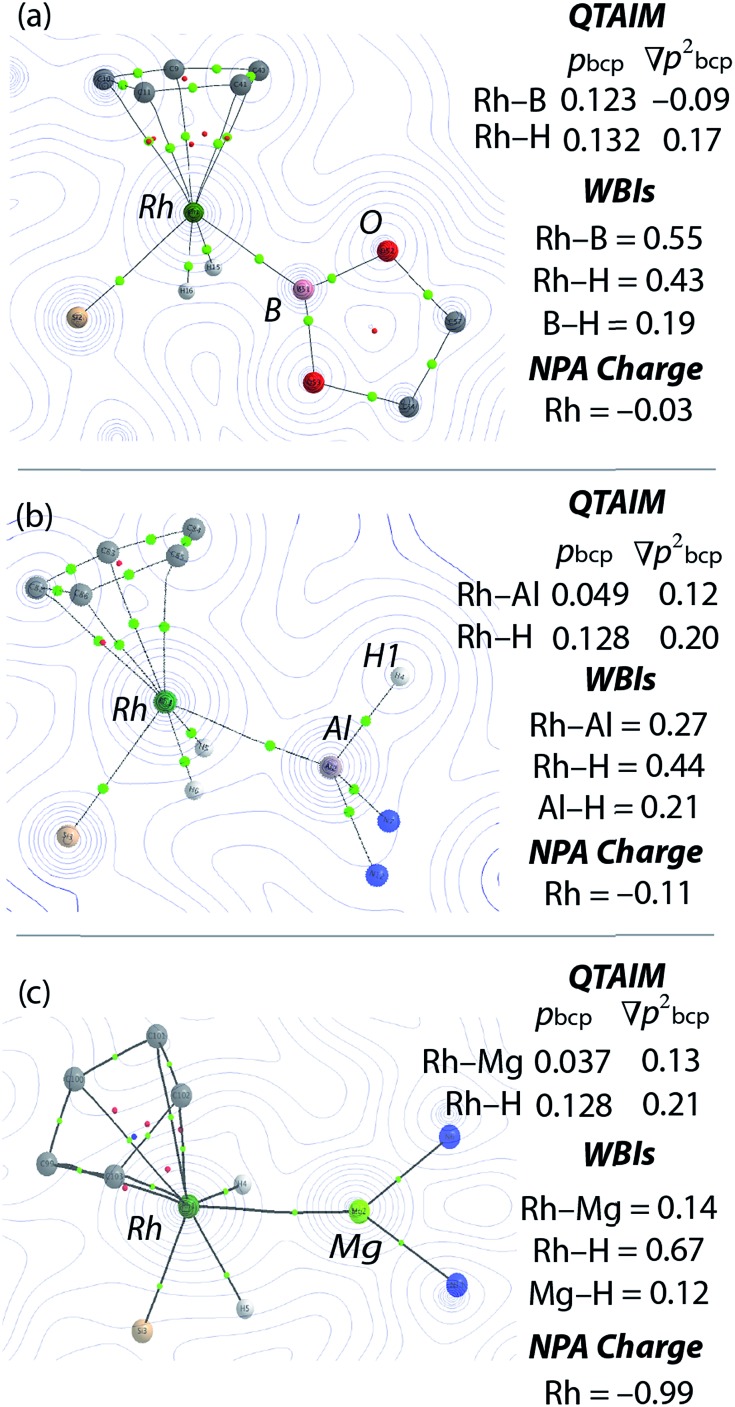
Electron density contour plots with over-layered calculated structures from QTAIM of (a) [Cp*Rh(H)_2_(Bpin)(SiEt_3_)] presented in the {RhBO} plane, (b) **1** presented in the {RhAlH1} plane and (c) **3** presented in the {RhMgN} plane. For X–H bonds (X = Rh, B, Al, Mg) data are given as the mean, green dots are bond critical points, red dots ring critical points.

Two extreme bonding descriptions can be considered: a neutral rhodium complex containing a Rh–M bond (**A**) and a rhodiate complex in which a cationic main group fragment is stabilised by coordination to a rhodium anion (**B**). Both bonding descriptions are likely to be augmented by additional non-classical M···H interactions. As a result the assignment of a formal oxidation state has limited meaning. In order to verify the position of the hydride atoms and to gain a deeper insight into the bonding within the ground-state structures of **1–3** a series of gas-phase DFT calculations were conducted. For comparison, [Cp*Rh(H)_2_(Bpin)(SiEt_3_)] and [Cp*Rh(H)_2_(SiEt_3_)_2_] were also analysed by DFT methods (Fig. 2and 3).

**Fig. 4:**
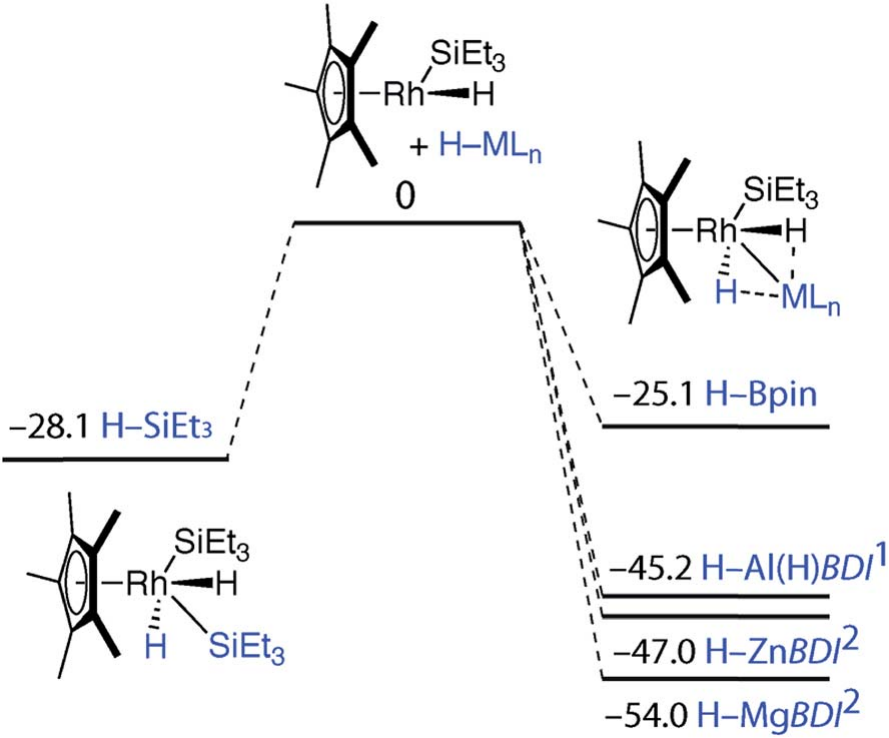
Potential energy surfaces from gas phase DFT calculations (Gibbs free energies, 298.15 K, values in kcal mol^–1^). All minima confirmed by frequency calculations.

NBO calculations revealed that, while the Rh–M Wiberg bond indices of **1**, **2** and **3** are lower than those of [Cp*Rh(H)_2_(SiEt_3_)_2_] and [Cp*Rh(H)_2_(Bpin)(SiEt_3_)], the Rh–H WBIs are consistent across the series (Fig. 4, ESI†). Suggestive of a weak interaction between the hydrides the main group element, the M···H WBIs for the series range from 0.12 to 0.21. The data are consistent with the aforementioned calculations on [Cp*Rh(H)_2_(SiEt_3_)_2_] which demonstrate a low energy barrier to hydride translation and formation of a weak Si···H bond. It has also been concluded that [Cp*Rh(H)_2_(Bpin)(SiEt_3_)] contains a weak B···H interaction as evidenced by the line-broadening of the hydride resonances due to ^1^ *J*_B–H_ coupling.^10^ It is worth noting that a series of related complexes, including [Cp*Rh(PMe_3_)(η^2^-HSiR_3_)(SiR_3_)]^+^ (R = Me, Et) and [CpRh(SiMe_3_)_2_(η^2^-HSiEt_3_)] have been formulated as σ-silane adducts based on the observation of significant ^1^ *J*_Si–H_ coupling constants > 20 Hz.^11e,f^

Our findings were further underscored by quantum theory atoms-in-molecules (QTAIM) calculations, which in all cases reveal bond critical points (BCPs) between Rh and M, Rh and H and not between M and H (Fig. 4, ESI†). The limitation of this method in detecting very weak interactions has been highlighted previously and it remains likely that the partial M···H bonds suggested by the NBO analysis and the spectroscopic data are valid.^19^ The NPA charges on the bridging hydride atoms are small throughout the series. While the charge on Rh is also small for the B, Si and Al complexes it becomes significant for the Zn and Mg analogues taking values of –1.01 and –0.99 respectively (Fig. 4 and ESI†). In combination the experimental and theoretical data suggest that the bonding description lies between the two extremes of **A** and **B**. The rhodiate structure **B** becoming more important for the Zn and Mg analogues of the series and the neutral rhodium structure **A** more important for the Si and B analogues of the series. The Al complex provides an intermediate case. Broadly, the spectrum of reactivity may be correlated with the electronegativity difference between hydrogen and the main group element: Δ*χ*_p_ = 0.16 (B), 0.30 (Si), 0.55 (Zn), 0.59 (Al), 0.89 (Mg).

To gain insight into the mechanism of bond activation and to provide support for an elimination-addition process, additional DFT calculations were conducted. The reductive elimination of triethylsilane from [Cp*Rh(H)_2_(SiEt_3_)_2_] to form [Cp*Rh(H)(SiEt_3_)] was found to be energetically accessible. A transition state could not be located for this transformation. Scanning the potential energy surface by gradually increasing a Rh···Si distance within [Cp*Rh(H)_2_(SiEt_3_)_2_] revealed that reductive elimination of H–SiEt_3_ to form the reactive intermediate is energetically uphill but without an easily identifiable maxima corresponding to a transition state. The addition of the M–H bond to the resulting 16-electron intermediate was calculated to be increasingly exergonic across the series Si ∼ B < Al ∼ Zn < Mg (Fig. 4). While the current data cannot rule out an associative type mechanism, such as σ-complex assisted metathesis or an interchange mechanism such as σ-bond metathesis, without invoking ring-slippage of the cyclopentadienyl ligand, these pathways remain unlikely to originate from the coordinatively saturated 18-electron complex [Cp*Rh(H)_2_(SiEt_3_)_2_].

**Scheme 2:**
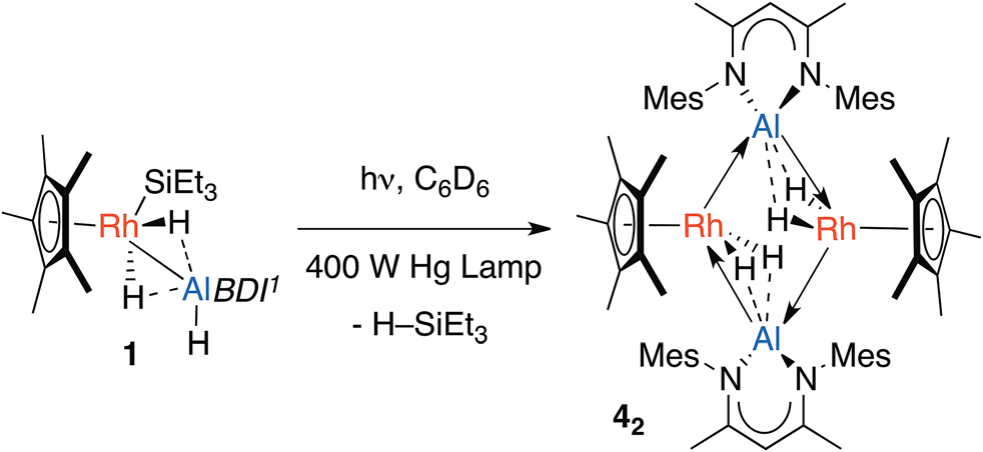
Photochemical elimination of HSiEt_3_ from **1** to form **4_2_**. Dotted lines represent the weak interaction between H and Al, these could also be represented by a half-arrow from the hydride to Al.^6^

The calculations suggest the addition of Al–H, Zn–H and Mg–H bonds to [Cp*Rh(H)(SiEt_3_)] may be non-reversible, while the addition of Si–H and B–H bonds is reversible (Fig. 4). In line with these findings, Hartwig and co-workers have previously demonstrated that [Cp*Rh(H)_2_(SiEt_3_)(Bpin)] undergoes competitive reductive elimination of both the silane, H–SiEt_3_, and the borane, H–Bpin upon heating in the presence of P(*p*-tol)_3_.^10^ While in the current case extended thermolysis of **1–3** led only to slow decomposition, photolysis of **1** for 6 h using a 400 W Hg lamp gave clean formation of **4_2_**, a product resulting from the selective elimination of H–SiEt_3_ from the heterobimetallic precursor (Scheme 2). Complex **4_2_** could also be generated directly under the same conditions by photolysis of a 1 : 1 mixture of *BDI*AlH_2_ and [Cp*Rh(H)_2_(SiEt_3_)_2_].

**Fig. 5:**
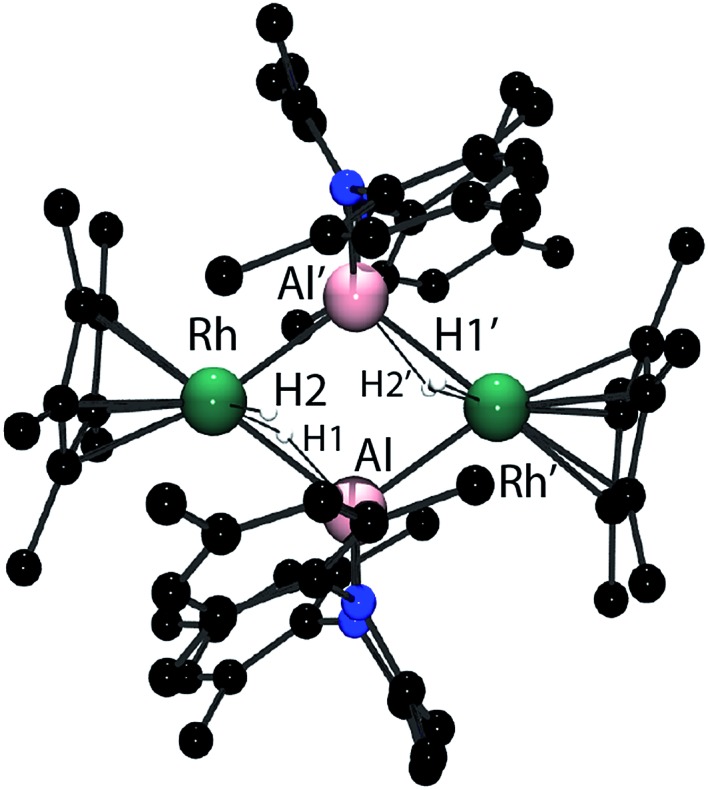
The crystal structure of **4_2_**. Selected bond angles (^o^) and bond lengths (Å). Rh–Al 2.5282(7), Rh–Al’ 2.4973(7), Al–Rh’–Al 70.97(2), Rh–Al–Rh’ 108.94(2). Hydrides were located within the Fourier difference map.

In the solid-state **4_2_** exists as a dimer of Rh–Al units forming a Rh_2_Al_2_H_4_ core with a point of inversion at the centre of the heterometallacycle (Fig. 5). Although still within the sum of the covalent radii, the Rh–Al distances of 2.4973(7) and 2.5282(7) Å are both longer than that found in **1**. The long Al···H distances of 1.99(3) and 2.07(3) Å and short Rh–H distances of 1.58(3) and 1.52(3) Å, suggest that, similar to **1**, hydride transfer occurs from Al to Rh. In this instance, no terminal hydride remains on Al and two Rh–Al bonds are formed per Al centre. The hydrides were located in the difference map and their position has been verified by DFT calculations on a truncated model of **4_2_** (see ESI†). Infrared spectroscopy supports the formulation and a Rh–H stretch is observed at 1988 cm^–1^.

In solution the hydride resonances are observed as a doublet at *δ* = –15.38 ppm (^1^ *J*_Rh–H_ = 44.2 Hz, fwhm = 7.8 Hz). The ^103^Rh NMR chemical shift of *δ* = –878 ppm is significantly different to those found in the series **1–3** (*vide supra*) and is consistent with an extreme change in the chemical and magnetic environment at rhodium upon elimination of triethylsilane. A series of DOSY experiments were conducted to probe the nuclearity of **4_2_** in solution. We have previously demonstrated that at 303 K in toluene-d^8^ solution, monomeric β-diketiminate aluminium and zinc hydrides possess diffusion coefficients in the range 0.967–0.944 × 10^–9^ m^2^ s^–1^ (*r*_solution_ = 4.1 Å) and that upon coordination to a copper(i) fragment the values decreases to 0.775 × 10^–9^ m^2^ s^–1^ (*r*_solution_ = 5.3 Å).^8*g*^ In the current case, the diffusion coefficient of **4_2_** of 0.591 × 10^–9^ m^2^ s^–1^ in C_6_D_6_ at 298 K is distinct from previous data and gives a solution hydrodynamic radius (*r*_solution_ = 6.2 Å) that is a similar magnitude to that calculated for the Rh_2_Al_2_ structure from the solid state data (*r*_solid_ = 7.3 Å).^20^ The data support the retention of the tetrametallic unit in solution.

Triethylsilane elimination from **1** is expected to lead to a monomeric heterobimetallic [Cp*Rh(H)_2_(Al*BDI* ^1^)] (**4**), which could be described as a Rh(iii) complex supported by an Al(i) ligand, or a Rh(i) complex with an η^2^;η^2^-bound aluminium dihydride (see ESI, Fig. S22†). The formation of a strongly σ-donating *BDI* ^1^Al: ligand within the coordination sphere of Rh would be expected to generate an extremely electron rich rhodium centre and dimerisation of **4** may occur by formation of an additional donor–acceptor interaction from a filled d-orbital on Rh to the partially vacant 3p-orbital on Al. Consistent with this argument in the X-ray structure of **4_2_** the diketiminate ligands on Al pucker away from planarity and exist in a conformation in which the orbital overlap between the lone-pairs on nitrogen and the Al(3p) orbital would be disrupted. Hence, we tentatively propose that the Al fragment in **4_2_** acts as an LZ-type ligand rather than an X_2_-type ligand and that the monomeric unit **4** could be formulated with a terminal Al(i) ligand (see ESI†).^6^

Aldridge and co-workers have provided computational data to support a similar Al(i)/Co(iii) formulation in the cationic fragment of the cobalt complex [(OC)_3_Co(μ-H)_2_Al*BDI* ^*2*^][Co(CO)_4_]. This latter species also contains an additional donor–acceptor interaction in the form of coordination of an oxygen based lone pair from the anionic [Co(CO)_4_] unit to Al. Furthermore, the generation of a gallium(i) ligand from the spontaneous transition metal mediated dehydrogenation of a β-diketiminate supported gallium dihydride has been reported by the same group.^8d,8e^ Two pathways could explain the generation of **4** from **1**, either a 1,2-elimination of H–SiEt_3_ from across the Rh–Al bond or reductive elimination of H–SiEt_3_ from the Rh metal centre followed by α-migration of the terminal hydride on Al to Rh. Based on the current data we cannot discriminate these mechanisms.

## Conclusions

We have demonstrated the addition of aluminium, zinc and magnesium hydrides to a coordinatively unsaturated, 16-electron, Rh(iii) complex [Cp*Rh(H)(SiEt_3_)]. The reaction results in the cleavage of the M–H bond and the formation of a new Rh–M and Rh–H bond. While parallels may be drawn with an oxidative addition mechanism, only in the case of Al is this description valid. For Zn–H and Mg–H calculations reveal considerable charge localisation on rhodium and hence hydride transfer to generate an ate complex is a more apt description of the reaction. DFT calculations suggest that the addition of M–H (M = Al, Zn and Mg) bonds to the Rh(iii) intermediate is non-reversible. This latter hypothesis has been supported by initial reactivity studies, which demonstrate the selective elimination of triethylsilane from a rhodium complex containing ligands derived from both a silane and an alane. We are continuing to study the unusual heterobimetallics reported herein as catalysts and reagents for difficult bond transformations.
