# Supplementary material for: Addition of aluminium, zinc and magnesium hydrides to rhodium(iii)
Source: Chem Sci. 2015 Jul 3;6(10):5617–22. doi: 10.1039/c5sc01309g (PMC5510526; doi:10.1039/c5sc01309g)
Supplement: Supplementary file 1 [file SC-006-C5SC01309G-s001.pdf]

Electronic Supplementary Material (ESI) for Chemical Science.
This journal is © The Royal Society of Chemistry 2015

Supporting information for:

Addition of Aluminum, Zinc and Magnesium Hydrides to Rhodium(III)

Olga Ekkert[a], *Andrew J. P. White*[a], *Harold Toms*[b], *Mark R. Crimmin*[a*]

[a]Department of Chemistry, Imperial College London, South Kensington, London, SW7 2AZ, U.K.

[b]The School of Biological and Chemical Sciences, Queen Mary, University of London, Mile End Road, London E1 4NS, U.K.

Table of Contents

**1**	**General Experimental**	S3
**2.1**	**Synthesis of Rh–M hetereobimetallics (M = Al, Zn, Mg)**	S4
**2.2**	**X-ray Crystallography Data**	S9
**2.3**	**Multinuclear and VT NMR studies**	S12
**2.4**	**DFT Studies**	S18
**3**	**References**	S24
**4**	**Z-Matrices**	S25

1 General Experimental

All manipulations were carried out under standard Schlenk-line or glovebox techniques under an inert atmosphere of nitrogen. A MBRAUN Labmaster glovebox was employed operating under an inert atmosphere of concentrations of H_2O and O_2 below 0.1 ppm. Glassware was dried for 12 hours at 120 °C prior to use. C_6D_6 and D_8-toluene were stored over molecular sieves for twelve hours before using. All other solvents were dried using a Grubbs type solvent purification system. ^{1}H, ^{13}C, ^{29}Si NMR experiments were run within Youngs Tap NMR tubes on 400 MHz or 500 MHz BRUCKER machines. NOSEY, DOSY, HSQC, and DEPT-135 experiments were run on a 400 MHz or 500 MHz NMR machine. The majority of ^{1}H spectra were recorded from + 30ppm to - 20ppm. Spectra were referenced to known solvent peaks. NMR analysis was conducted in Topspin or MestReNova with baseline corrections applied to spectra. Infrared spectra were obtained from solids on an ATR cell. CHN Analysis were performed by Dr. Stephen Boyer of London Metropolitan University. Rh-complexes $[Cp^*RhCl(\mu\text{-}Cl)]_2$[1] and $[Cp^*Rh(SiEt_3)_2(H)_2]$[2], main-group hydrides BDI^1AlH_2, BDI^2ZnH and BDI^2MgH were prepared according to the literature procedures.[3-5] All other reagents were purchased from Sigma-Aldrich and were used without further purification. DOSY data were processed within MestreNova using the Bayesian DOSY transformation, while solid-state radii of **4₂** was estimated from the DFT optimised structure using the Volume keyword in Gaussian09.

The $^{1}H\{^{103}Rh\}$ HMQC experiments were recorded at 303K on a Bruker AVANCE 600 MHz spectrometer and referenced assuming a frequency of 18.964 MHz for $Rh(acac)_3$. 2048 complex points were acquired in the ^{1}H dimension with a spectral width of 15015 Hz. 256 t1 increments were collected in the indirect (^{103}Rh) dimension with a spectral width of 56835 Hz, 8 scans per increment. The acquisition time was 0.068s with a relaxation delay of 1s, the total experiment time being 9m 28s. A gradient ratio of 70:30:43.19 was used. The data was apodized with a sine-bell function and presented in magnitude mode. The $\pi/2$ pulses for ^{1}H and ^{103}Rh were 8 μs and 44 μs respectively, a 12 μs pulse for 103Rh being used in the experiment. A value of 37 Hz was assumed for the ^{1}H-^{103}Rh coupling giving a 1/2J delay for the experiment of 0.0135s.

2.1.1 Synthesis of Rh-M heterobimetallics (M = Al, Zn, Mg)

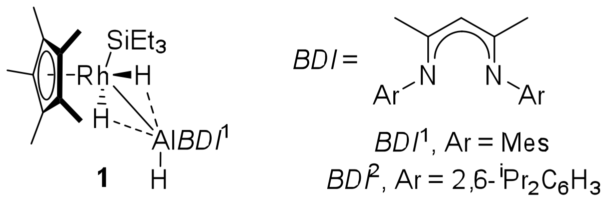

In a glovebox, $[Cp^*Rh(SiEt_3)_2(H)_2]$ (80 mg, 0.168 mmol, 1 equiv.) was dissolved in toluene (2 mL) and a toluene solution (2 mL) containing BDI^1AlH_2 (55 mg, 0.151 mmol, 0.9 equiv.) was added at room temperature. The reaction mixture was stirred at 100 °C for 20 h. The solvent was removed under vacuum and the residue was suspended in *n*-hexane (4 mL). After filtration *n*-hexane was evaporated, the crude product was dissolved in $O(SiMe_3)_2$ (2 mL) and stored at -35 °C to give a crystalline, light orange product of complex **1**. The solid was isolated by decantating of the mother liquor and then dried under vacuum to give product **1** in 40% yield (43 mg, 0.06 mmol).

^{1}H NMR (400 MHz, D_8-toluene, 297 K): δ = 6.79 (s, 4H, *m*-CH^{Mes}), 5.24 (br, 1H, Al*H*), 4.97 (s, 1H, C*H*), 2.42, 2.39 (each s, each 6H, *o*-CH_3^{Mes}), 2.15 (s, 6H, *p*-CH_3^{Mes}), 1.67 (s, 15H, CH_3^{Cp*}), 1.51 (s, 6H, CH_3), 1.08 (m, 9H, SiCH$_2$CH_3), 0.94 (m, 6H, SiCH_2CH$_3$), -15.14 (d, $^1J_{RhH}$ = 40.2 Hz, 2H, Rh-*H*-Al).

T_1 (400 Hz) = ~0.9s (Rh-*H*-Al).

^{13}C NMR (101 MHz, D_8-toluene, 297 K): δ = 169.4 (*C*CH$_3$), 141.9 (*i*-C^{Mes}), 135.5 (*p*-C^{Mes}), 134.8, 134.5 (*o*-C^{Mes}), 129.9 (d, *J* = 5.8 Hz, *m*-C^{Mes}), 98.7 (d, J_{RhC} = 3.1 Hz, C^{Cp*}), 98.4 (*C*H), 23.4 (*C*H$_3$), 21.3 (*p*-*C*H_3^{Mes}), 20.6 (*o*-*C*H_3^{Mes}), 12.8 (br, Si*C*H$_2$CH$_3$), 10.8 (br, SiCH$_2$*C*H$_3$), 10.6 (*C*H_3^{Cp*}).

^{27}Al{^{1}H} NMR (104 MHz, D_8-toluene, 297 K): δ = 144.7 ($\nu_{1/2}$ ~ 700 Hz).

^{29}Si{^{1}H} NMR (80 MHz, D_8-toluene, 297 K): δ = 38.6.[a]

^{103}Rh{^{1}H} NMR (19 MHz, C_6D_6, 303 K): δ = -1570.[b]

Elemental analysis calc. for $C_{39}H_{62}AlN_2RhSi$: C, 65.34; H, 8.72; N, 3.91 found C, 65.19; H, 8.59; N, 3.86.

ATR-IR (solid, cm^{-1}): 1966 (Rh-*H*-Al), 1709 (Al*H*).

---

[a] Signal observed from ^{1}H, ^{29}Si{^{1}H}HMBC NMR experiment.
[b] Signal observed from ^{1}H,^{103}Rh{^{1}H}HMQC NMR experiment.

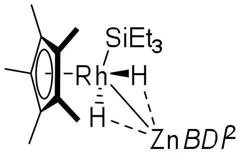

2

In a glovebox, [Cp*Rh($SiEt_3$)$_2$(H)$_2$] (80 mg, 0.168 mmol, 1equiv.) was dissolved in toluene (2 mL) and a toluene solution (3 mL) containing *BDI*^{2}ZnH (73 mg, 0.151 mmol, 0.9 equiv.) was added at room temperature. The respective mixture was stirred at 100 °C for 20 h. The solvent was removed under vacuum and the residue was suspended in *n*-hexane (4 mL). After filtration the crude product was then stored at -35 °C to give a crystalline solid of complex **2**. The product was isolated by decanting the mother liquor and then dried under vacuum to give colorless crystals of compound **2** (31 mg, 0.037 mmol, 25%).

^{1}H NMR (500 MHz, C_6D_6, 297 K): δ = 7.23, 7.17, 7.13 (each m, each 2H, ArC*H*), 4.73 (s, 1H, C*H*), 3.59, 3.37 (each sept., $^3J_{HH}$ = 6.68 Hz, each 2H, C$H^{i\text{-}Pr}$), 1.61 (s, 15H, CH_3^{Cp*}), 1.61 (s, 6H, CH_3), 1.57, 1.39, 1.25, 1.15 (each d, $^3J_{HH}$ = 6.68 Hz, each 6H, C$H_3^{i\text{-}Pr}$), 1.25 (m, 9H, SiCH$_2$CH_3), 1.04 (m, 6H, SiCH_2CH$_3$), -14.28 (d, $^1J_{RhH}$ = 34.6 Hz, 2H, Rh-*H*-Zn).

T_1 (500 MHz) = ~0.9s (Rh-*H*-Zn).

^{13}C NMR (126 MHz, C_6D_6, 297 K): δ = 168.0 (*C*CH$_3$), 145.8 (*i*-$C^{i\text{-}Pr}$), 143.9, 142.2 (*o*-$C^{i\text{-}Pr}$), 125.9, 124.7, 123.6 (Ar*C*H), 97.3 (d, J_{RhC} = 3.4 Hz, C^{Cp*}), 95.9 (*C*H), 29.3, 28.3 ($CH^{i\text{-}Pr}$), 26.8, 25.3, 25.0, 24.4 ($CH_3^{i\text{-}Pr}$), 24.5 (*C*H$_3$), 12.8 (br, Si*C*H$_2$CH$_3$), 11.6 (CH_3^{Cp*}), 11.2 (br, SiCH$_2$*C*H$_3$).

^{29}Si{^{1}H} NMR (99 MHz, C_6D_6, 297 K): δ = 31.6 (d, $^1J_{RhSi}$ = 20.6 Hz).

^{103}Rh{^{1}H} NMR (19 MHz, C_6D_6, 303 K): δ = -1743.[b]

Elemental analysis calc. for $C_{45}H_{73}N_2RhSiZn$: C, 64.46; H, 8.78; N, 3.34 found C, 64.30; H, 8.84; N, 3.46.

ATR-IR (solid, cm^{-1}): 1959 (Rh-*H*-Zn).

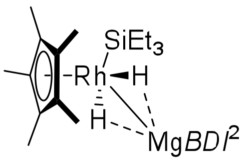

3

In a glovebox, $[Cp^*Rh(SiEt_3)_2(H)_2]$ (100 mg, 0.212 mmol, 1equiv.) was dissolved in toluene (2 mL) and a toluene solution (3 mL) containing *BDI*²MgH (85 mg, 0.191 mmol, 0.9 equiv.) was added at room temperature. The respective mixture was stirred at 80 °C for 4 d. The solvent was removed under vacuum and the residue was suspended in *n*-hexane (5 mL). After filtration the crude product was then stored at -35 °C and crystallization of a colorless compound was observed. Product **3** was isolated in 28% (42 mg, 0.053 mmol) yield as a colorless crystalline compound after decantation of the mother liquor and drying under vacuum.

^{1}H NMR (500 MHz, D_8-toluene, 233 K): δ = 7.16 (m, 4H, ArC*H*), 7.10 (m, 2H, ArC*H*), 4.70 (s, 1H, C*H*), 3.42, 3.30 (each sept., $^3J_{HH}$ = 6.71 Hz, each 2H, $CH^{i\text{-}Pr}$), 1.64 (s, 15H, $CH_3^{Cp^*}$), 1.57 (s, 6H, CH_3), 1.57, 1.40, 1.27, 1.13 (each d, $^3J_{HH}$ = 6.71 Hz, each 6H, $CH_3^{i\text{-}Pr}$), 1.29 (m, 9H, $SiCH_2CH_3$), 1.05 (m, 6H, $SiCH_2CH_3$), -15.91 (d, $^1J_{RhH}$ = 40.5 Hz, 2H, Rh-*H*-Mg).

T_1 (500 MHz, 297 K) = ~1s (Rh-*H*-Mg).

^{13}C NMR (126 MHz, D_8-toluene, 233 K): δ = 169.9 (*C*CH$_3$), 145.2 (i-$C^{i\text{-}Pr}$), 143.7, 141.9 (o-$C^{i\text{-}Pr}$), 126.1, 125.1, 123.9 (Ar*C*H), 96.3 (d, J_{RhC} = 3.0 Hz, C^{Cp^*}), 95.7 (*C*H), 29.8, 28.7 ($CH^{i\text{-}Pr}$), 26.8 (CH_3), 25.4, 25.4, 24.8, 24.5 ($CH_3^{i\text{-}Pr}$), 12.7 (br, $SiCH_2CH_3$), 12.1 (br, $SiCH_2CH_3$), 11.9 ($CH_3^{Cp^*}$).

^{29}Si{^{1}H} NMR (99 MHz): δ = 34.7.[a]

^{103}Rh{^{1}H} NMR (19 MHz, C_6D_6, 303 K): δ = -1540.[b]

Due to fast decomposition, within hours, in both the solid and solution, even under an atmosphere of N_2, attempts to acquire elemental analysis or mass spec data on **3** failed.

ATR-IR (solid, cm^{-1}): 1929 (Rh-*H*-Mg).

2.1.2 Synthesis of Rh-Al heterobimetallic dimer

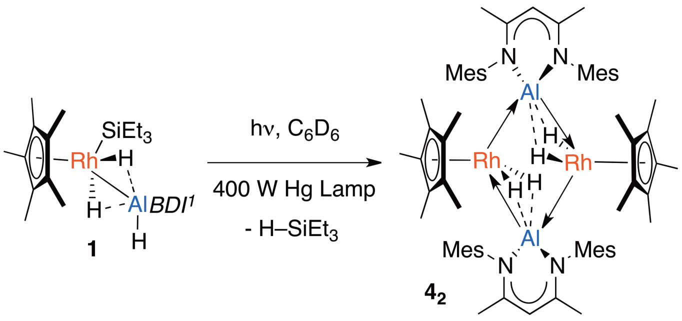

Method a: In a glovebox, [$Cp^*Rh(SiEt_3)_2(H)_2$] was dissolved in toluene and a toluene solution containing *BDI*^{1}AlH_2 was added at room temperature. The reaction mixture was transferred to a J Youngs NMR tube (either borosilicate or quartz), placed 5 cm from an immersion lamp and irradiated (400 W Hg lamp) at room temperature for 6 h. The solvent was concentrated under vacuum, the residue was filtered and then stored at room temperature to give a crystalline, orange product of complex **4**$_2$. The solid was isolated by decantating the mother liquor and then dried under vacuum to give product **4**$_2$.

Method b: In a glovebox, complex **1** was dissolved in toluene. The reaction mixture was transferred to a J Youngs NMR tube (either borosilicate or quartz), placed 5 cm from an immersion lamp. The mixture was irradiated (400 W Hg lamp) at room temperature for 6 h. Workup and isolation of compound **4**$_2$ were done using method a.

Following method a, compound **4**$_2$ was isolated in 24% yield (27 mg, 0.04 mmol) using $Cp^*Rh(SiEt_3)_2H_2$ (100 mg, 0.212 mmol, 1 equiv.) and *BDI*^{1}AlH_2 (70 mg, 0.191 mmol, 0.9 equiv.).

^{1}H NMR (400 MHz, C_6D_6, 297 K): δ = 7.02, 6.86 (each s, each 2H, *m*-CH^{Mes}), 5.08 (s, 1H, C*H*), 2.44, 2.32 (each s, each 6H, *o*-CH_3^{Mes}), 2.27 (s, 6H, *p*-CH_3^{Mes}), 1.67 (s, 15H, CH_3^{Cp*}), 1.55 (s, 6H, CH_3), -15.38 (d, $^1J_{RhH}$ = 44.2 Hz, 2H, Rh-*H*-Al).

^{13}C NMR (101 MHz,C_6D_6, 297 K): δ = 168.1 (*C*CH_3), 145.5 (*i*-C^{Mes}), 136.4 (*p*-C^{Mes}), 134.0, 133.6 (*o*-C^{Mes}), 130.5, 129.0 (*m*-C^{Mes}), 101.7 (*C*H), 97.3 (C^{Cp*}), 24.4 (*C*H_3), 22.0, 20.9 (*o*-*C*H_3^{Mes}), 21.6 (*p*-*C*H_3^{Mes}), 11.7 (*C*H_3^{Cp*}).

^{27}Al{^{1}H} NMR (104 MHz, C_6D_6, 297 K): δ = 140 ($\nu_{1/2}$ ~ 700 Hz).

^{103}Rh{^{1}H} NMR (19 MHz, C_6D_6, 303 K): δ = -878.[b]

Elemental analysis calc. for $C_{66}H_{92}Al_2N_4Rh_2$: C, 65.99; H, 7.72; N, 4.66 found C, 66.12; H, 7.79; N, 4.58.

ATR-IR (solid, cm^{-1}): 1988 (Rh-*H*-Al).

2.2 X-ray Crystallographic Data

The X-ray crystal structure of 1

Crystal data for **1**: $C_{39}H_{62}AlN_2RhSi \cdot {}^1/_2(C_6H_{14})$, M = 759.97, monoclinic, $P2_1/n$ (no. 14), a = 10.8994(3), b = 20.7161(5), c = 18.9857(4) Å, β = 92.160(2)°, V = 4283.80(17) Å^3, Z = 4, D_c = 1.178 g cm^{-3}, μ(Mo-Kα) = 0.476 mm^{-1}, T = 173 K, colourless tabular needles, Agilent Xcalibur 3E diffractometer; 8589 independent measured reflections (R_{int} = 0.0247), F^2 refinement,[6] R_1(obs) = 0.0374, wR_2(all) = 0.0898, 6798 independent observed absorption-corrected reflections [$|F_o| > 4\sigma(|F_o|)$, $2\theta_{max}$ = 57°], 453 parameters. CCDC 1047853.

The Al–H and Al–H–Rh hydrogen atoms in the structure of **1** were located from Δ*F* maps and refined freely.

The X-ray crystal structure of 2

Crystal data for **2**: $C_{45}H_{73}N_2RhSiZn$, M = 838.42, triclinic, P-1 (no. 2), a = 10.3195(7), b = 11.6531(5), c = 19.8109(10) Å, α = 77.860(4), β = 75.973(5), γ = 73.269(5)°, V = 2188.0(2) Å^3, Z = 2, D_c = 1.273 g cm^{-3}, μ(Mo-Kα) = 0.984 mm^{-1}, T = 173 K, colourless platy needles, Agilent Xcalibur 3E diffractometer; 8562 independent measured reflections (R_{int} = 0.0260), F^2 refinement,[6] R_1(obs) = 0.0349, wR_2(all) = 0.0656, 6692 independent observed absorption-corrected reflections [$|F_o| > 4\sigma(|F_o|)$, $2\theta_{max}$ = 56°], 477 parameters. CCDC 1047854.

The Zn–H–Rh hydrogen atoms in the structure of **2** were located from Δ*F* maps and refined freely.

The X-ray crystal structure of 3

Crystal data for **3**: $C_{45}H_{73}MgN_2RhSi$, M = 797.36, triclinic, P-1 (no. 2), a = 10.3775(4), b = 11.6602(7), c = 19.8540(9) Å, α = 77.684(4), β = 75.691(4), γ = 73.192(4)°, V = 2202.2(2) Å^3, Z = 2, D_c = 1.202 g cm^{-3}, μ(Mo-Kα) = 0.460 mm^{-1}, T = 173 K, pale yellow platy needles, Agilent Xcalibur 3E diffractometer; 8666 independent measured reflections (R_{int} = 0.0250), F^2 refinement,[6] R_1(obs) = 0.0359, wR_2(all) = 0.0733, 7414 independent observed absorption-corrected reflections [$|F_o| > 4\sigma(|F_o|)$, $2\theta_{max}$ = 57°], 477 parameters. CCDC 1047855.

The Mg–H–Rh hydrogen atoms in the structure of **3** were located from Δ*F* maps and refined freely.

The X-ray crystal structure of $\mathbf{4_2}$

Crystal data for $\mathbf{4_2}$: $C_{66}H_{92}Al_2N_4Rh_2{\cdot}C_6H_6$, M = 1279.32, monoclinic, $C2/c$ (no. 15), a = 17.4233(3), b = 16.5193(3), c = 23.4839(4) Å, β = 104.9583(16)°, V = 6530.10(18) Å^3, Z = 4 (C_2 symmetry), D_c = 1.301 g cm^{-3}, μ(Cu-Kα) = 4.672 mm^{-1}, T = 173 K, orange blocks, Agilent Xcalibur PX Ultra A diffractometer; 6297 independent measured reflections (R_{int} = 0.0272), F^2 refinement,[6] R_1(obs) = 0.0308, wR_2(all) = 0.0813, 5458 independent observed absorption-corrected reflections [$|F_o| > 4\sigma(|F_o|)$, $2\theta_{max}$ = 148°], 380 parameters. CCDC 1056989.

The structure of $\mathbf{4_2}$ shows the complex to have crystallographic C_2 symmetry about an axis that passes through the middle of the Al_2Rh_2 ring and bisects the Al1···Al1A and Rh1···Rh1A vectors. The two unique Al–H–Rh hydrogen atoms were located from ΔF maps and refined freely. The C(41)-based benzene solvent molecule was found to be disordered across an inversion centre. Two unique orientations were identified of *ca*. 33 and 17% occupancy, with the operation of the centre of symmetry generating two further orientations of the same occupancies. The geometries of the two unique orientations were idealised, the thermal parameters of adjacent atoms were restrained to be similar, and all of the atoms were refined isotropically.

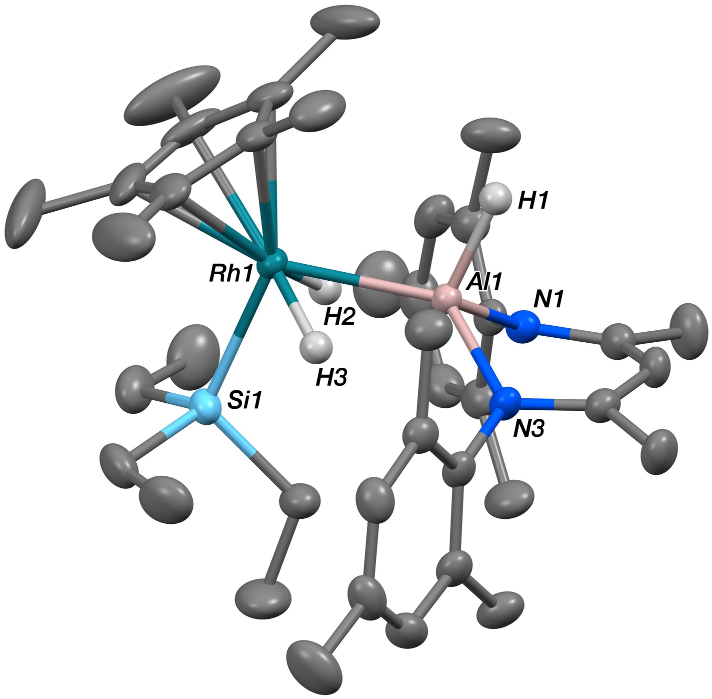

Figure S1 The crystal structure of **1** (50% probability ellipsoids).

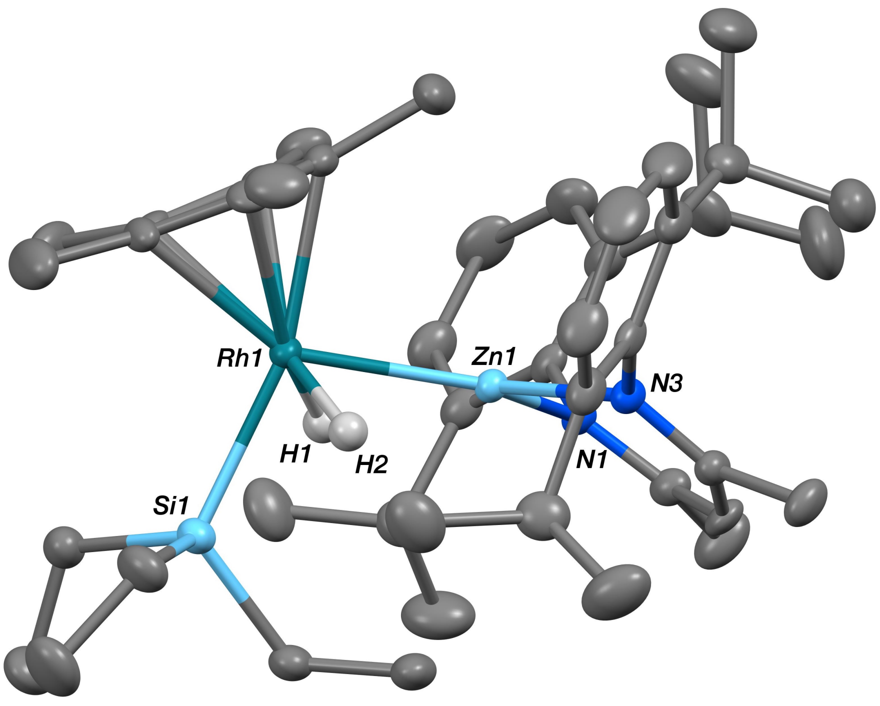

Figure S2 The crystal structure of **2** (50% probability ellipsoids).

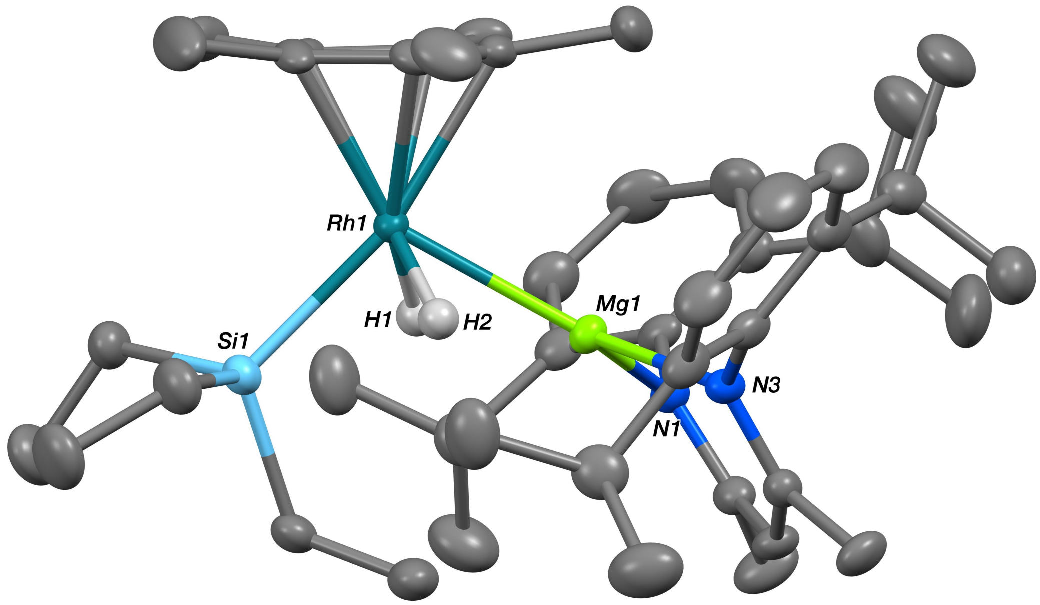

Figure S3 The crystal structure of **3** (50%

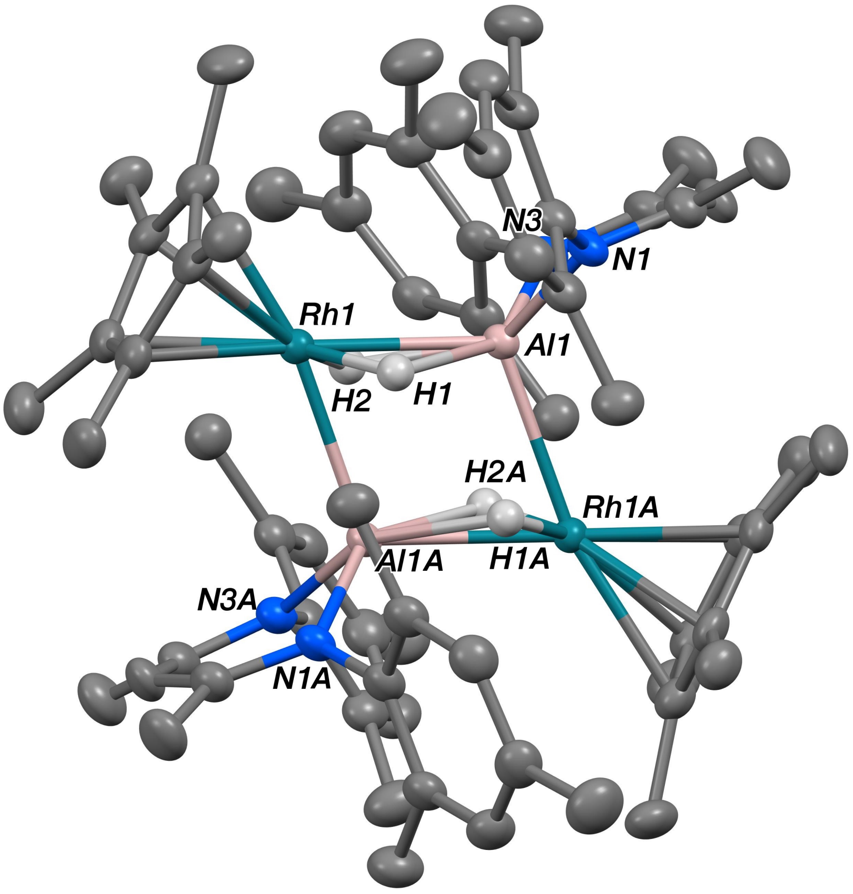

Fig. S4 The crystal structure of the C_2-symmetric complex $\mathbf{4_2}$ (50% probability ellipsoids).

2.3 Multinuclear and VT NMR studies

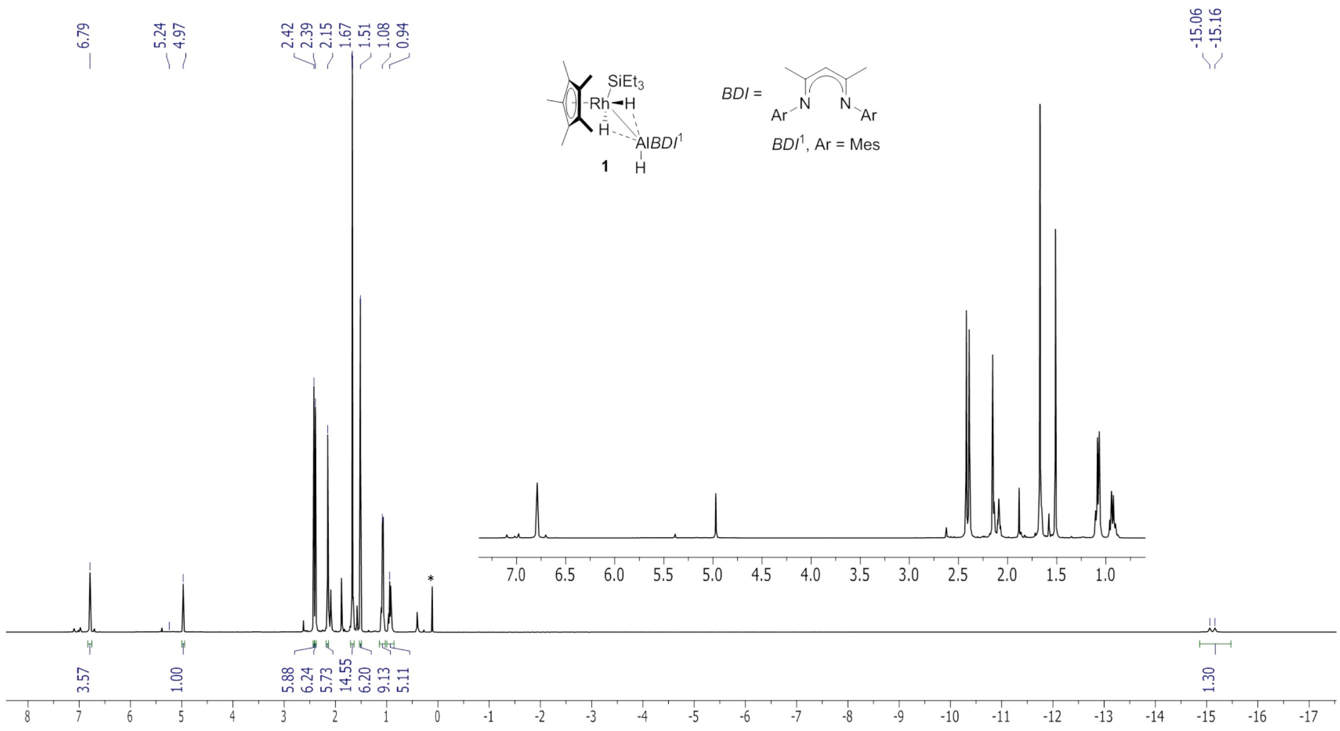

Figure S5. ^{1}H NMR (400 MHz, D_8-toluene, 297 K) of compound **1** (*$O(SiMe_3)_2$).

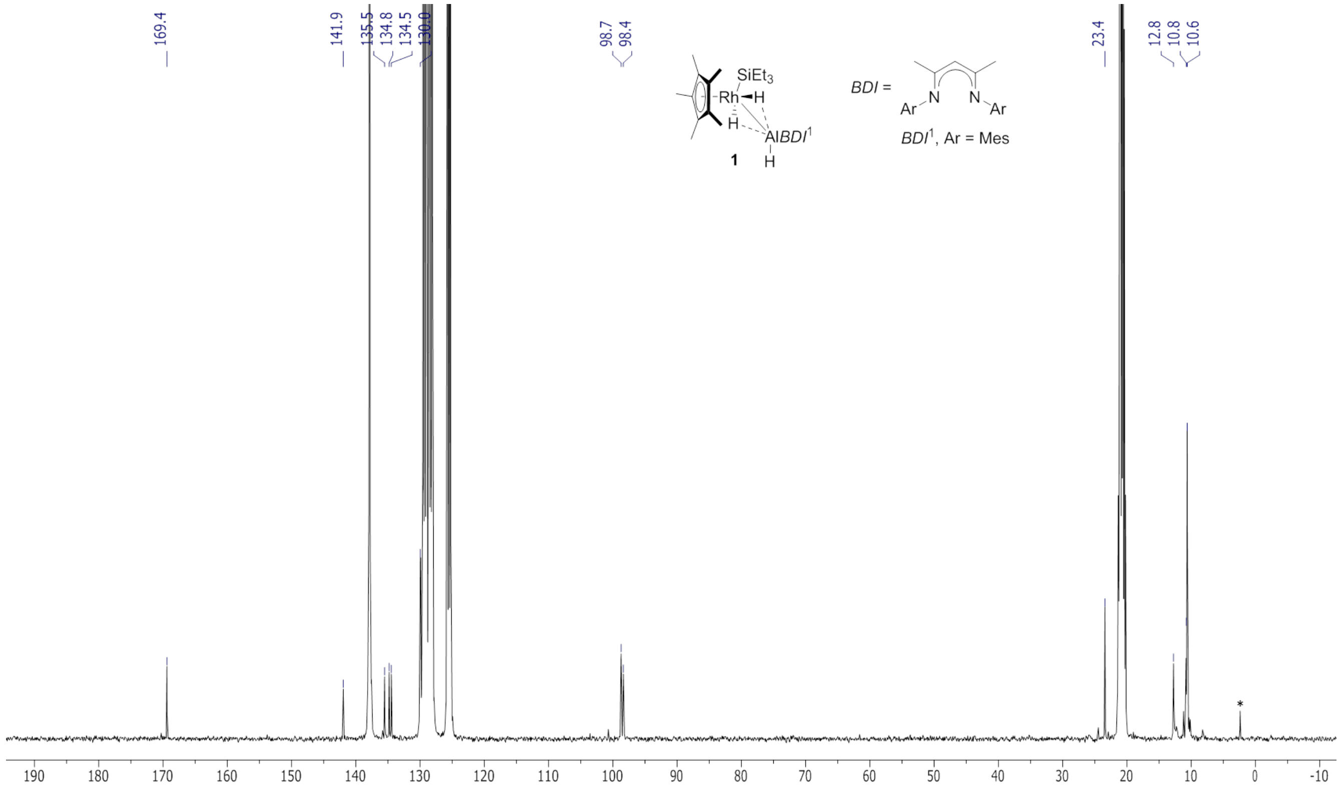

Figure S6. ^{13}C NMR (101 MHz, D_8-toluene, 297 K) of compound **1** (*$O(SiMe_3)_2$).

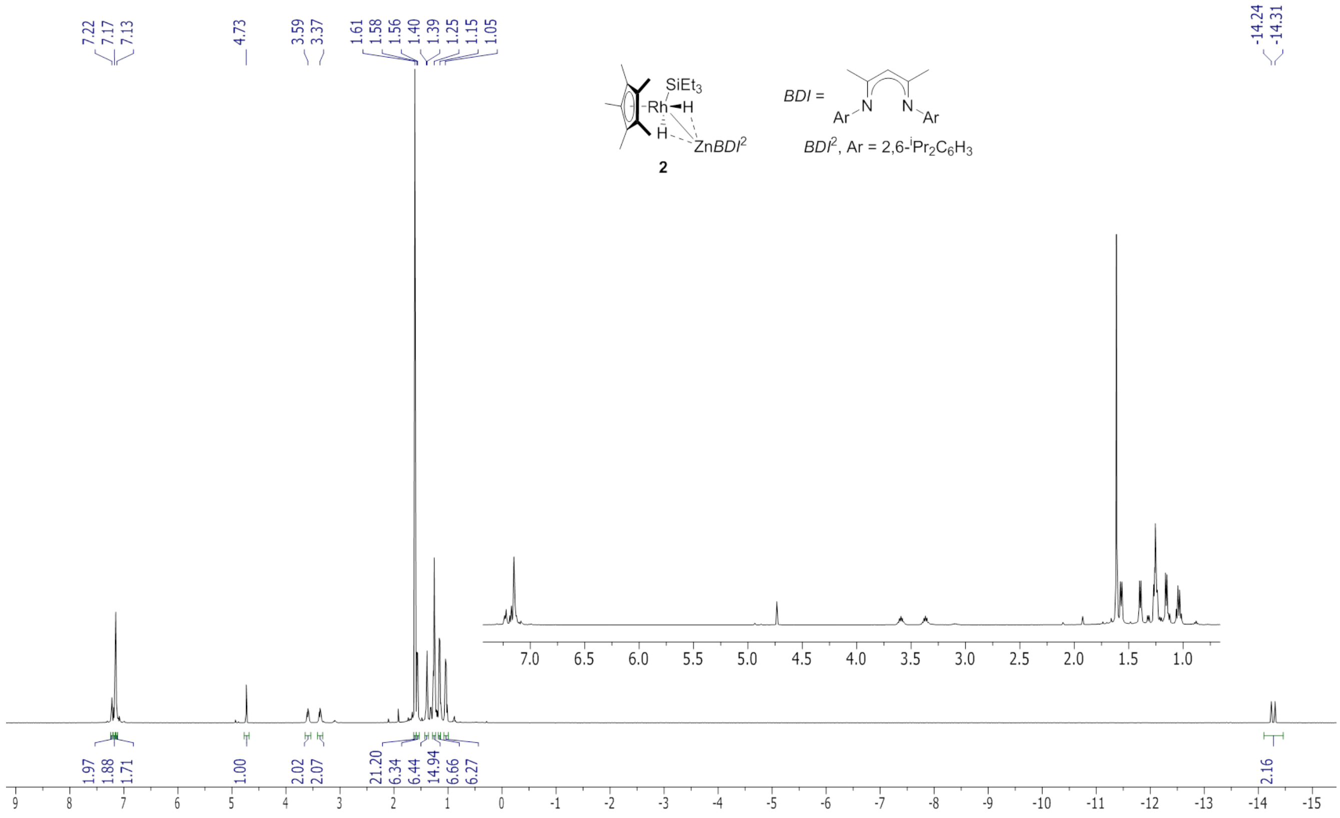

Figure S7. ^{1}H NMR (500 MHz, C_6D_6, 297 K) of compound **2**.

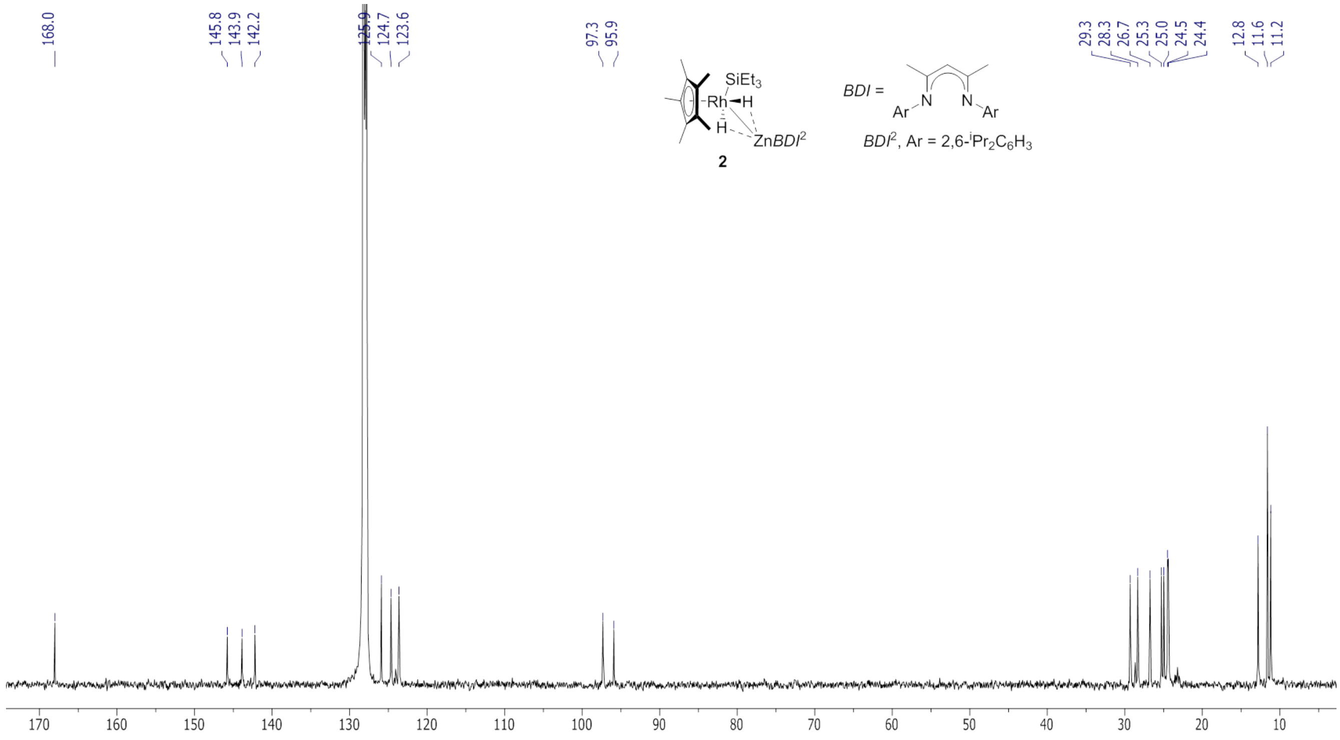

Figure S8. ^{13}C NMR (126 MHz, C_6D_6, 297 K) of compound **2.**

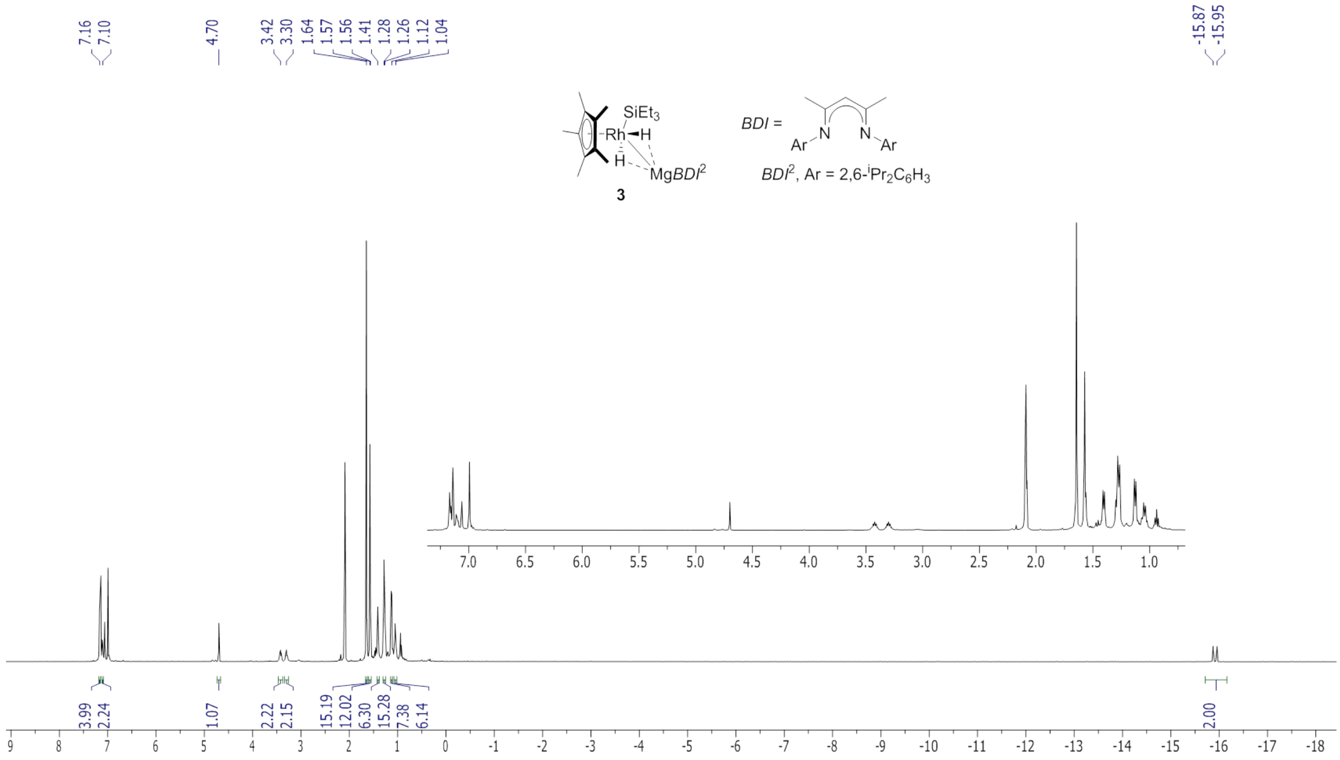

Figure S9. ^{1}H NMR (500 MHz, D_8-toluene, 233 K) of compound **3**.

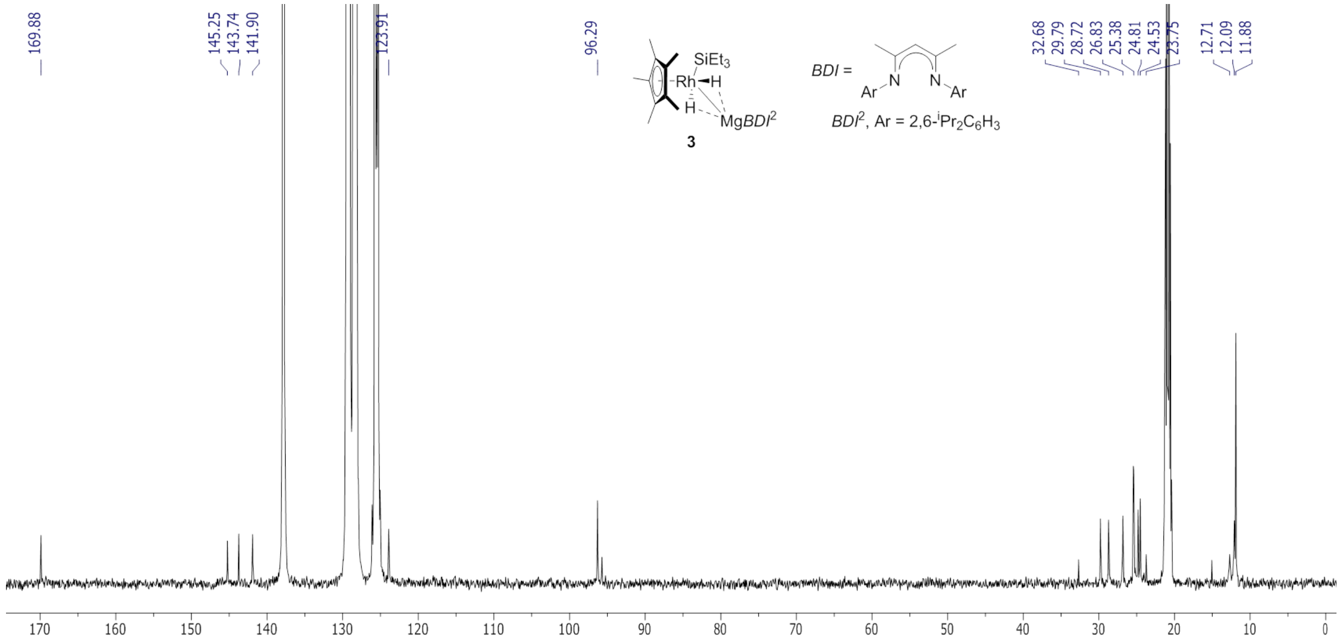

Figure S10. ^{13}C NMR (126 MHz, D_8-toluene, 233 K) of compound **3**.

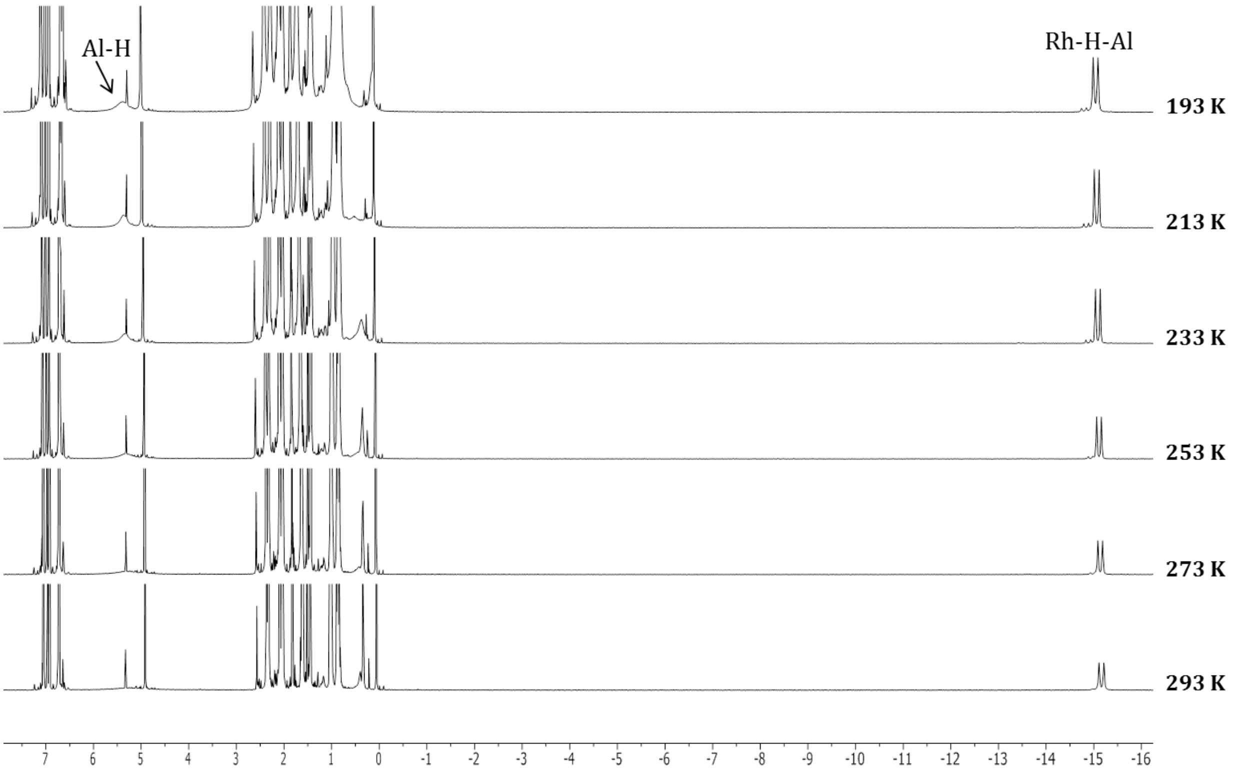

Figure S11. ^{1}H NMR (400 MHz, D_8-toluene) of compound **2**.

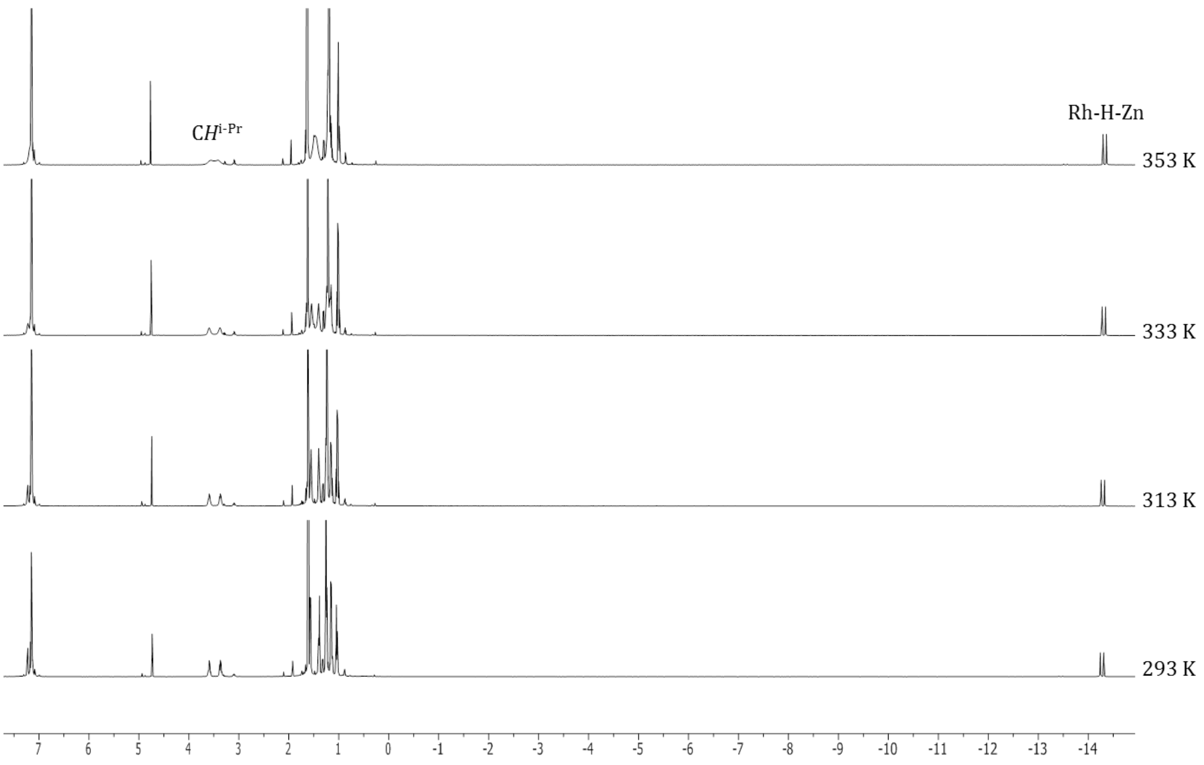

Figure S12. ^{1}H NMR (500 MHz, C_6D_6) of compound **3**.

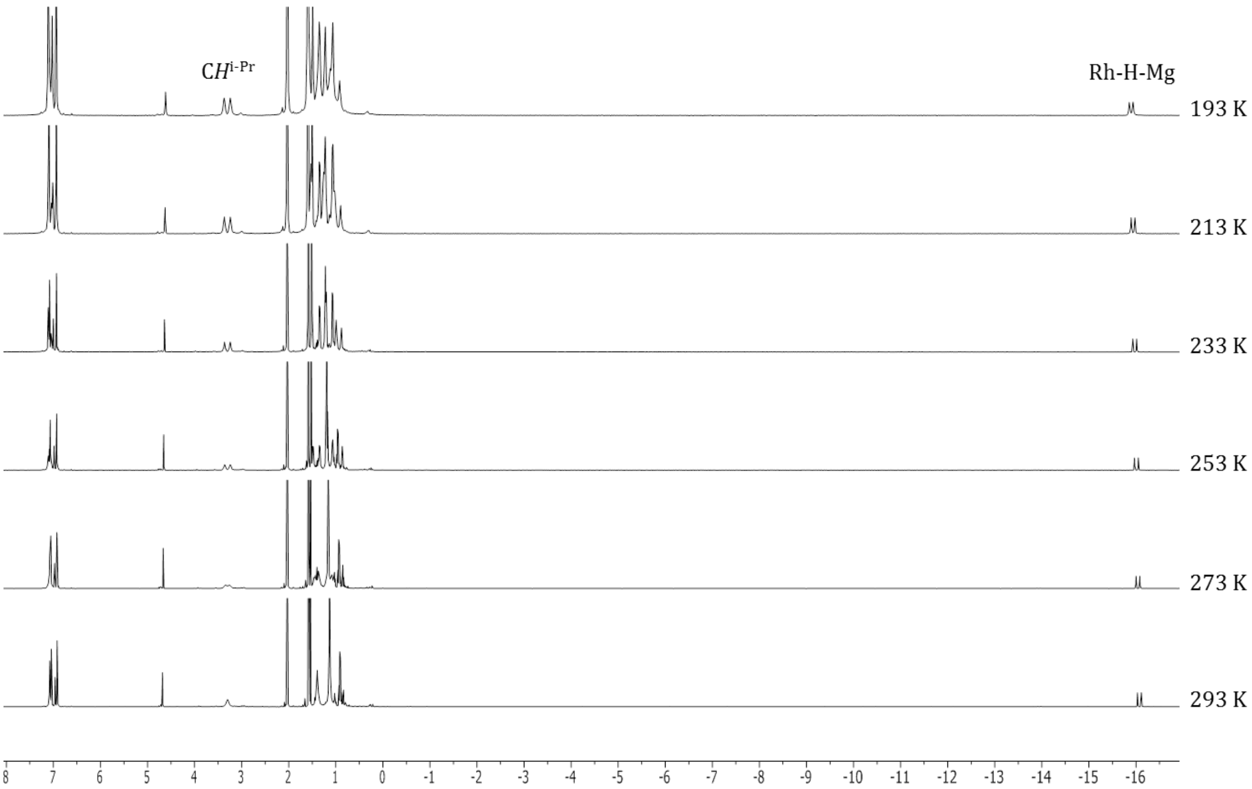

Figure S13. ^{1}H NMR (500 MHz, D_8-toluene) of compound **4**.

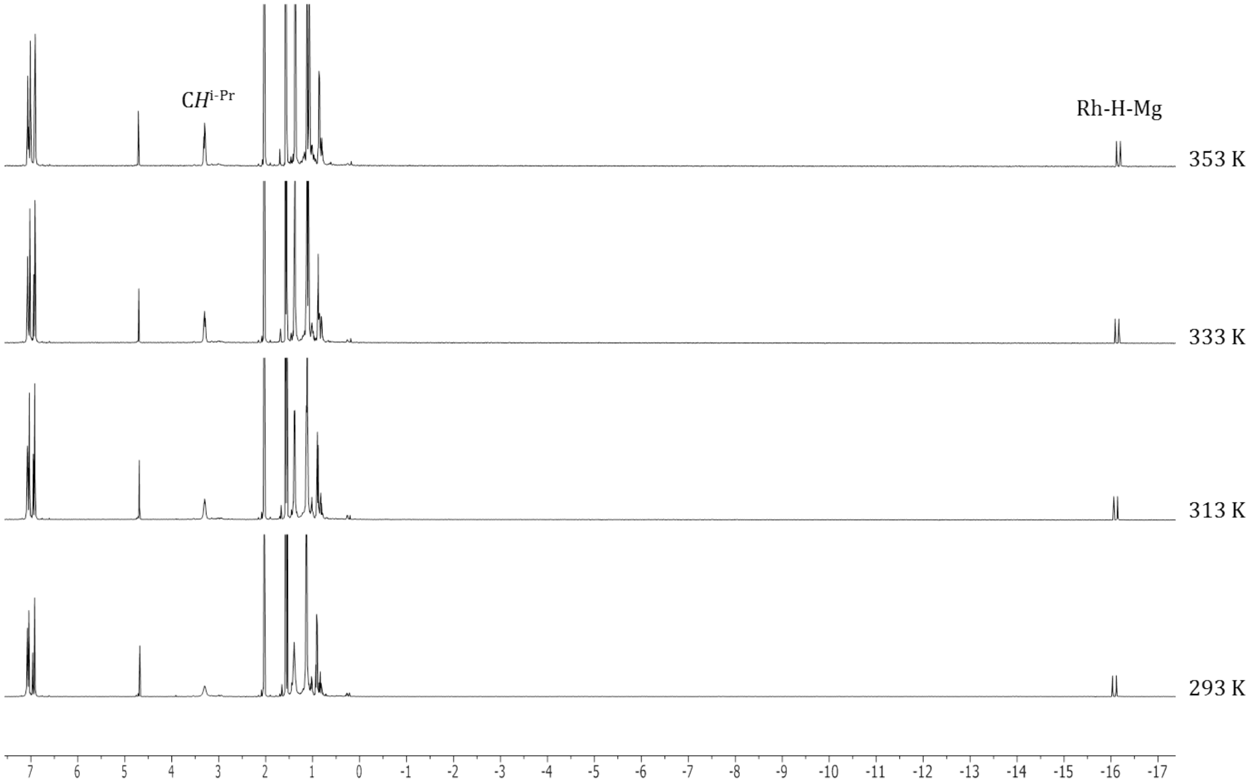

Figure S14. ^{1}H NMR (500 MHz, D_8-toluene) of compound **4**.

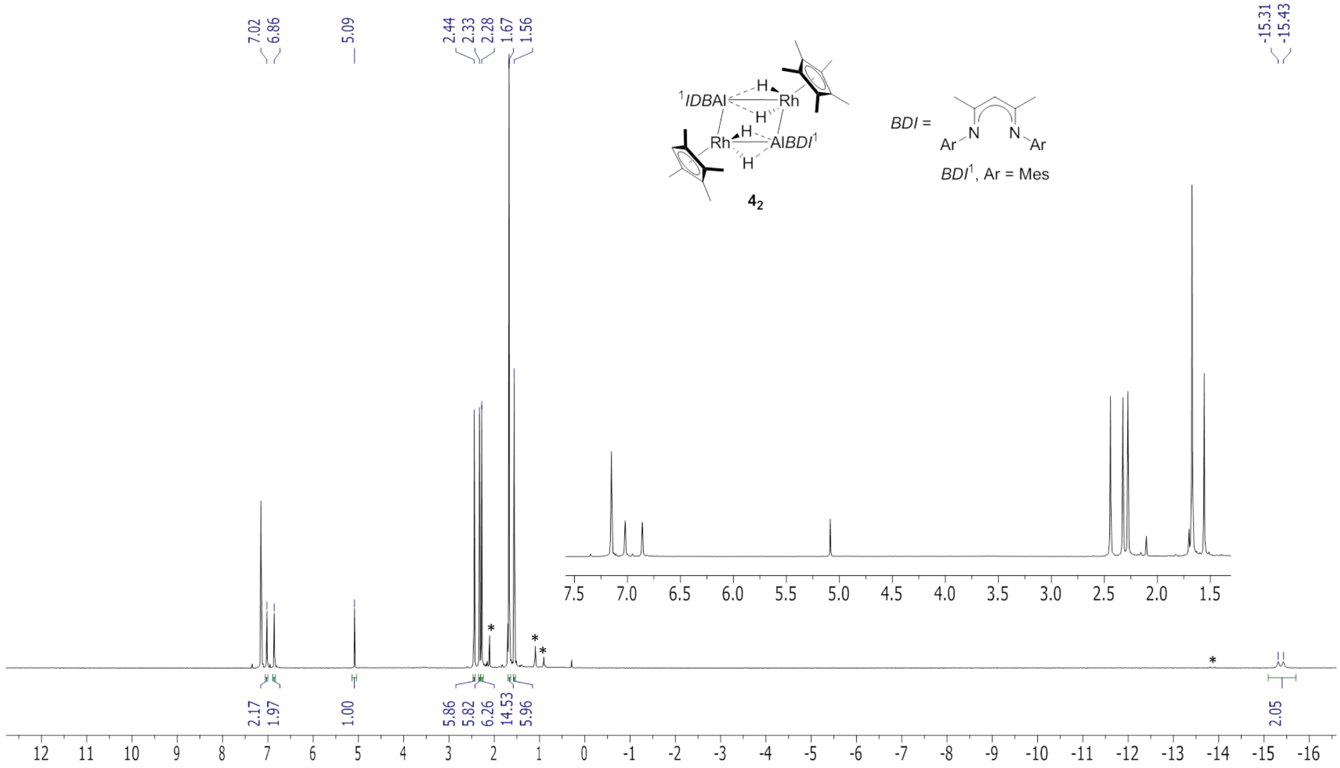

Figure S15. ^{1}H NMR (400 MHz, C_6D_6, 297 K) of compound **4_2** [(*)Cp*Rh($SiEt_3$)$_2H_2$].

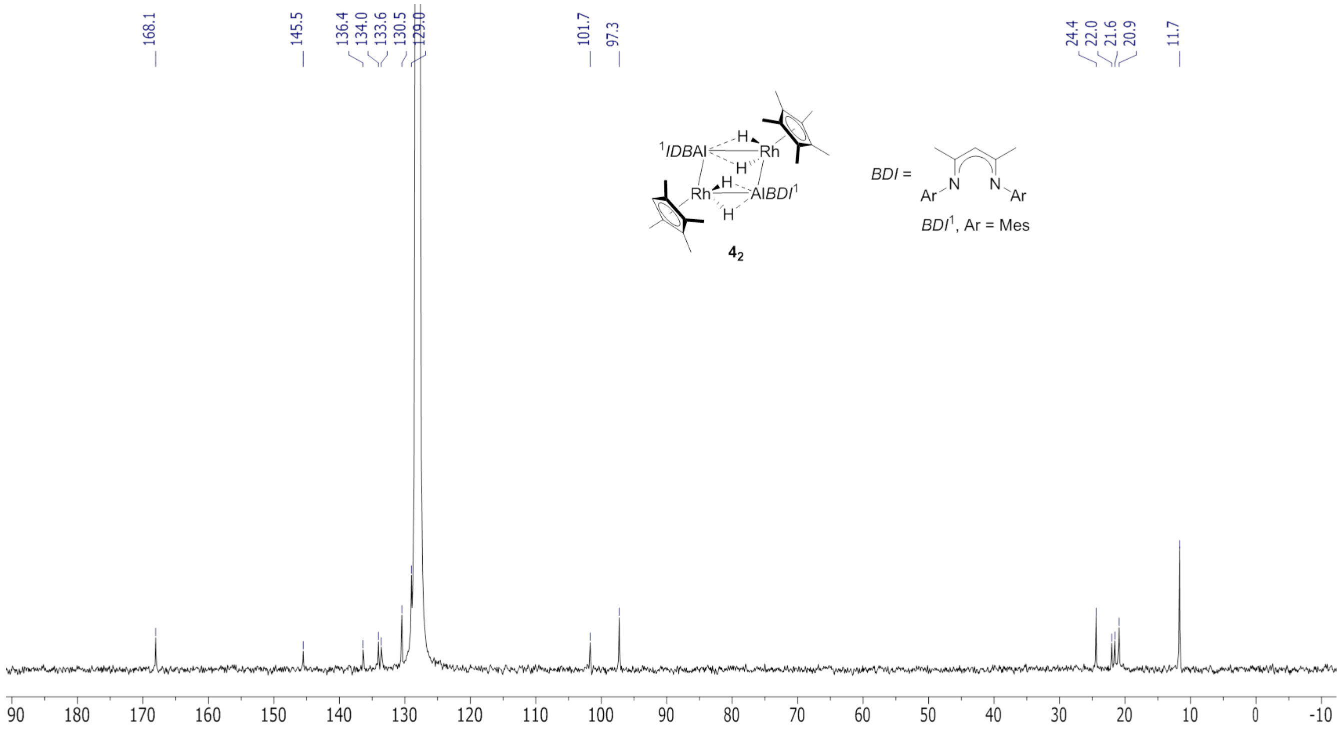

Figure S16. ^{13}C NMR (101 MHz, C_6D_6, 297 K) of compound **4_2**.

2.5 DFT Studies

2.5.1 General

Calculations were conducted in Gaussian09.[7] All minima were confirmed by frequency calculations and solid-state data were used as an input for the atom coordinates. NBO calculations were run using NBO v5.9 within g09. In all cases the geometries were compared against the solid-state data, an m062x functional and hybrid basis set 6,31G+(d,p) (C/H/N/Si/B) and Lanl2DZ (Al/Zn/Mg/Rh) was employed to probe the nature of bonding in **1-3**. Bader analysis was conducted on optimized geometries in the AIMALL package.[8]

2.5.2 Ground-States

For compounds **2** and **3** more than one minimum was observed computationally. Hence, conformationally distinct minima **2'** and **3'** were obtained and shown to be similar in energy to **2** and **3** respectively. These species differ by the geometry at the Et_3Si moiety, and demonstrate an agostic interaction of either the α-C–H or β-C–H position with Zn and Mg (Figure S1). Mimina **2'** and **3'** isomers were found to be higher in Gibbs free energy than **2** and **3** by 4.5 and 0.6 kcal mol^{-1} respectively and have not been considered in the discussion of the ground-states of the series of heterobimetallic complexes. For complex **4$_2$** a model **4$_2$-truncated** in which all the methyl groups were replaced by hydrogen atoms was used.

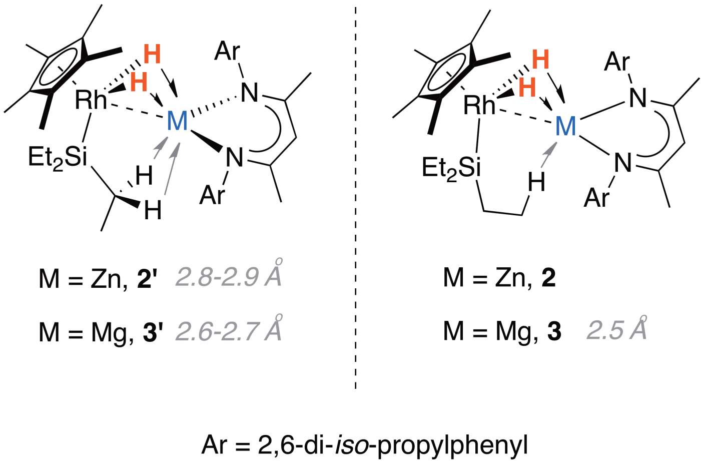

Figure S17. Comparison of Calculated Coordination modes **2/3** and **2'/3'.** M062x functional, 6,31G+(d,p)/Lanl2DZ basis-set. All minima confirmed by frequency calculations

	$Cp^*Rh(H)_2(SiEt_3)_2$		Maximum Error
	Exp.	Calc.	
**Rh-H**	1.58	1.57	0.6%
**Rh–Si**	2.379	2.393-2.397	0.8%
**Rh–M**			
**M---H**	2.27	2.25-2.33	2.6%
**Si–Rh–M**	107.90	109.24	1.2%

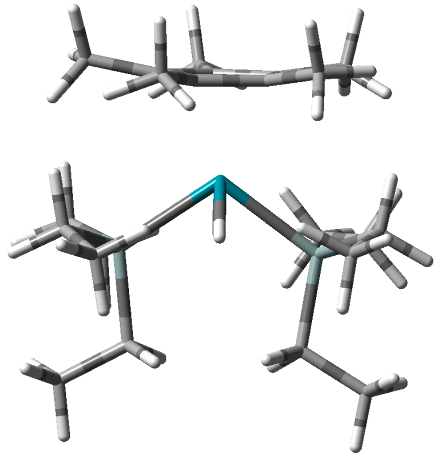

	$Cp^*Rh(H)_3(Bpin)$		Maximum Error
	Exp.	Calc.	
**Rh-H**	1.57-1.59	1.58	0.6%
**Rh–Si**	2.368	2.393	1.0%
**Rh–M**	2.038	2.00	1.9%
**M---H**	1.74-2.06	1.97-1.99	**13.6%**
**Si–Rh–M**	102.65	102.99	0.3%

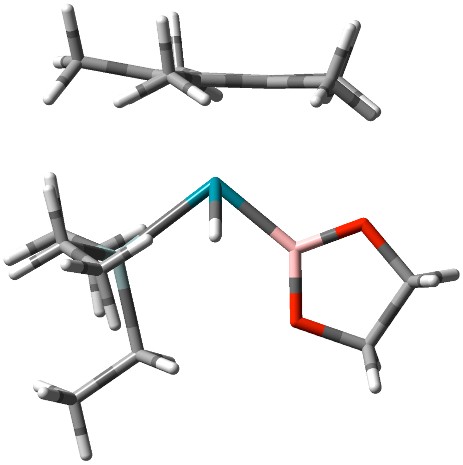

	**1**		Maximum Error
	Exp.	Calc.	
**Rh-H**	1.51-1.52	1.58-1.59	**5.0%**
**Rh–Si**	2.340	2.354	0.6%
**Rh–M**	2.453	2.456	0.1%
**M---H**	2.05-2.12	2.16-2.18	**6.0%**
**Si–Rh–M**	102.82	103.41	0.6%

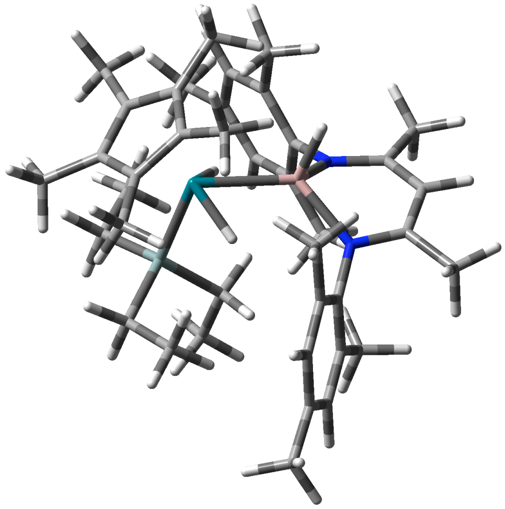

Figure S18a: Comparison of Calculated and Solid-State Data

	**2**		**Maximum Error**
	Exp.	Calc.	
**Rh-H**	1.42-1.56	1.57-1.59	**12.0%**
**Rh–Si**	2.357	2.358	0.0%
**Rh–M**	2.416	2.496	3.3%
**M---H**	2.14-2.17	2.09-2.25	**5.1%**
**Si–Rh–M**	106.86	105.06	1.7%

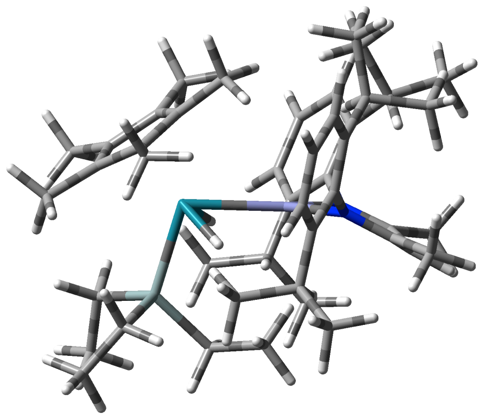

	**3**		**Maximum Error**
	Exp.	Calc.	
**Rh-H**	1.50-1.54	1.58-1.59	**5.7%**
**Rh–Si**	2.344	2.350	0.3%
**Rh–M**	2.505	2.518	0.5%
**M---H**	2.09	2.12-2.20	**5.0%**
**Si–Rh–M**	106.23	105.77	0.4%

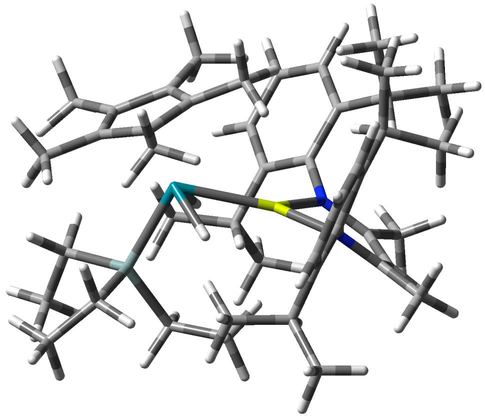

	**4_2-truncated**		**Maximum Error**
	Exp.	Calc.	
**Rh-H**	1.52-1.58	1.58-1.59	**4.4%**
**Rh–Al’**	2.582	2.481	4.1%
**Rh–Al**	2.497	2.467	1.2%
**M---H**	1.99-2.07	2.07-2.12	**6.1%**
**Al–Rh–Al’**	70.97	72.96	2.7%

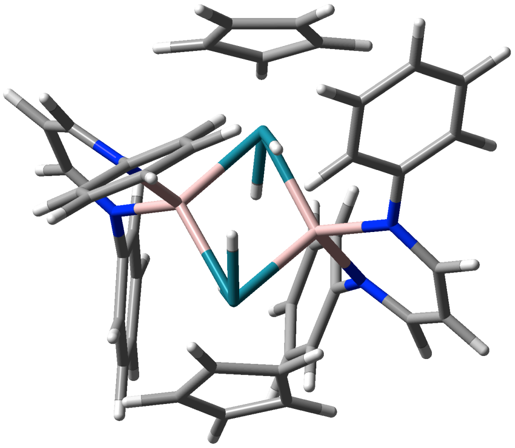

Figure S18b. Comparison of Calculated and Solid-State Data

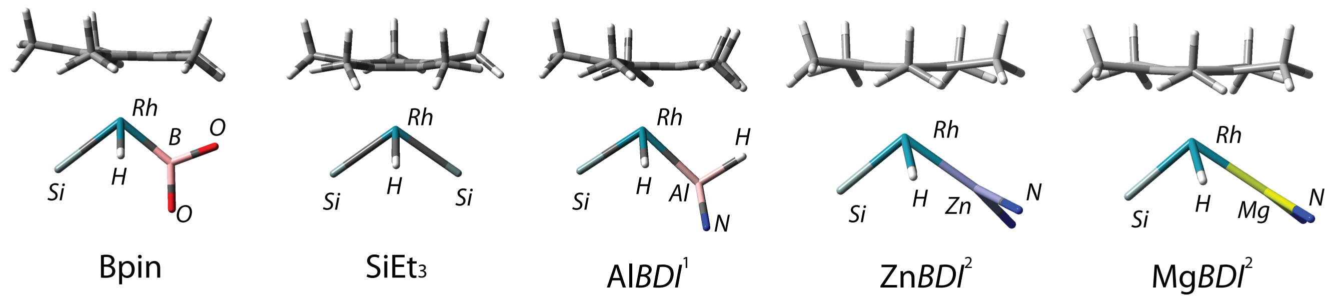

Figure S19. Side-on view of the calculated structure of the series [Cp*Rh(H)$_3$(Bpin)], [Cp*Rh(H)$_2$(SiEt$_3$)$_2$] and **1-3** reveals a slight bending of the main group atom towards the hydrides with decreasing electronegativity of the main group atom. M062x functional, 6,31G+(d,p)/Lanl2DZ basis-set. All minima confirmed by frequency calculations

Table S1. Wiberg Bond Indices (for entries 1-5, M = Si, B, Al, Zn, Mg respectively)

	Rh---M	Rh–H	M–H	Rh–Si
**Cp*Rh(H)$_2$(SiEt$_3$)$_2$**	0.41	0.44, 0.47	0.16, 0.18	0.41
**Cp*Rh(H)$_2$(Bpin)(SiEt$_3$)**	0.55	0.43, 0.44	0.19	0.41
**1**	0.27	0.44, 0.45	0.21, 0.22	0.47
**2**	0.23	0.65, 0.66	0.07, 0.08	0.58
**3**	0.14	0.67	0.11, 0.12	0.59

a – WBI of the terminal Al–H is 0.72

Table S2: Natural Population Analysis – Charge (for entries 1-5, M = Si, B, Al, Zn, Mg respectively)

	Rh	M	Si	Rh–**H**---M
**Cp*Rh(H)$_2$(SiEt$_3$)$_2$**	–0.06	+1.52		+0.03,+0.04
**Cp*Rh(H)$_2$(Bpin)(SiEt$_3$)**	–0.03	+0.94	+1.53	+0.06
**1**	–0.11	+1.46	+1.54	–0.03,–0.04^{a}
**2**	–1.01	+1.41	+1.64	+0.03,+0.08
**3**	–0.99	+1.53	+1.64	+0.01,+0.04

a – NPA charge on the terminal Al–H is –0.45

Table S3: QTAIM Analysis (for entries 1-5, M = Si, B, Al, Zn, Mg respectively)

	Rh–M ρ_{bcp}	Rh–M $\nabla\rho^2_{bcp}$	Rh–H_a ρ_{bcp}	Rh–$\mathbf{H}_a$ $\nabla\rho^2_{bcp}$	Rh–H_b ρ_{bcp}	Rh–$\mathbf{H}_b$ $\nabla\rho^2_{bcp}$
**Cp*Rh(H)$_2$(SiEt$_3$)$_2$**	0.071	–0.130	0.133	0.171	0.132	0.177
**Cp*Rh(H)$_2$(Bpin)(SiEt$_3$)**	0.123	–0.092	0.132	0.166	0.131	0.168
**1**	0.049	0.118	0.128	0.195	0.128	0.200
**2**	0.055	0.095	0.132	0.186	0.126	0.198
**3**	0.037	0.131	0.130	0.201	0.125	0.209

ρ_{bcp} (e bohr^{-3}); $\nabla\rho^2_{bcp}$ (e bohr^{-5})

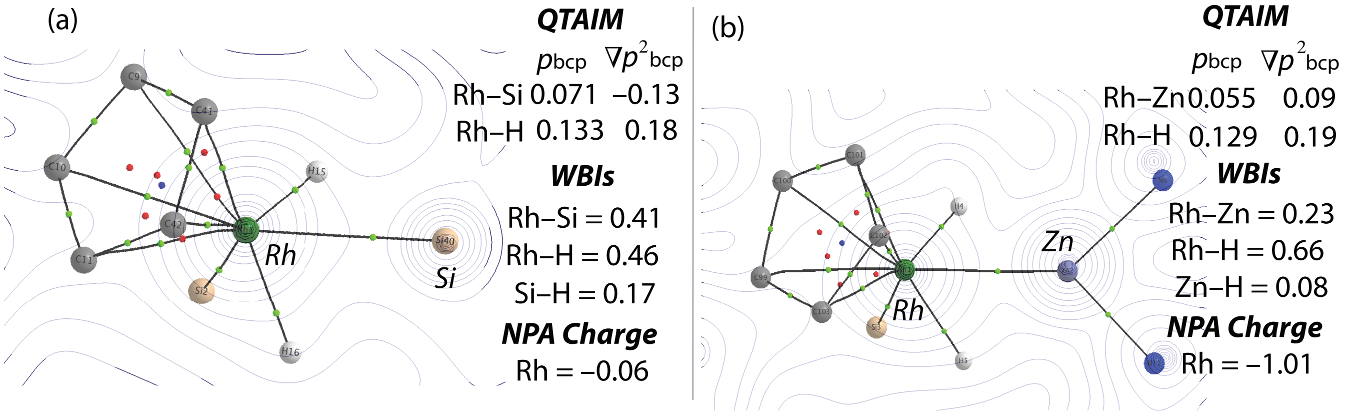

Figure S20: Electron density contour plots, WBIs and NPA charge for (a) [Cp*Rh(H)$_2$(SiEt$_3$)$_2$] presented in the {RhSiC} plane, and (b) **2** presented in the {RhZnN} plane. For X---H bonds (X = Rh, B, Al, Mg) data are given as the mean, green dots are bond critical points, red dots ring critical points

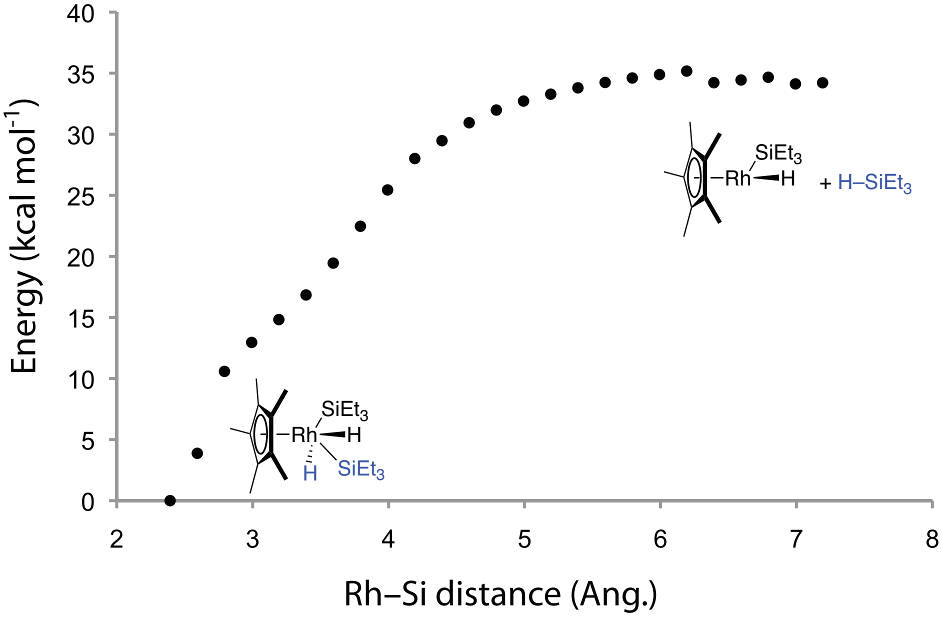

Figure S21: Relaxed PES of one Rh–Si vector of [Cp*Rh(H)$_2$(SiEt$_3$)$_2$].

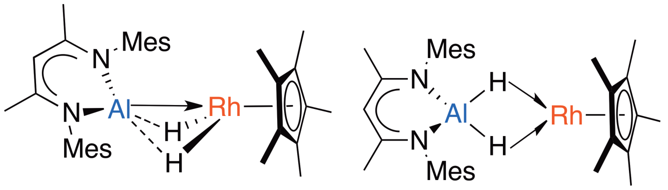

Table S4:

**4**	[*BDI*AlH$_2$]	[Cp*Rh]	ΔE_{AB}
-1505.28709712	-1006.02458277	-499.157902802	-0.104611548 (**-65.6 kcal mol^{-1}**)
**4**	[*BDI*Al:]	[Cp*RhH$_2$]	$\Delta E_{A'B'}$
-1505.28709712	-1004.80945315	-500.367568025	-0.110075945 (**-69.1 kcal mol^{-1}**)

Figure S22: The two extreme bonding descriptions in the monomeric unit **4**. Fragmentation analysis on **4**.[9] Each fragment was optimised and electronic energies were calculated by single point calculations. Values in Hartrees unless otherwise indicated.

3. References

1. Kang, J. W.; Moseley, K.; Maitlis, P. M. *J. Am. Chem. Soc.* **1969**, *91*, 5970.
2. (a) Fernandez, M.-J.; Maitlis, P. M. *J. Chem. Soc., Chem. Commun.* **1982**, 310. (b) Fernandez, M.-J.; Bailey, P. M.; Bentz, P. O.; Ricci, J. S.; Koetzle, T. F.; Maitlis, P. M. *J. Am. Chem. Soc.* **1984**, *106*, 5458.
3. Nako, A. E.; Gates, S. J.; White, A. J. P.; Crimmin, M. R. *Dalton Trans.* **2013**, *42*, 15199.
4. Spielmann, J.; Piesik, D.; Wittkamp, B.; Jansen, G.; Harder, S. *Chem. Commun.* **2009**, 3455.
5. Bonyhady, S. J.; Jones, C.; Nembenna, S.; Stasch, A.; Edwards, A. J.; McIntyre, G. J. *Chem. Eur. J.* **2010**, *16*, 938.
6. (a) SHELXTL, Bruker AXS, Madison, WI; (b) SHELX-97, G.M. Sheldrick, *Acta Cryst.*, **2008**, *A64*, 112-122; (c) SHELX-2013, http://shelx.uni-ac.gwdg.de/SHELX/index.php
7. Gaussian 09, Revision D.01, Frisch, M. J.; Trucks, G. W.; Schlegel, H. B.; Scuseria, G. E.; Robb, M. A.; Cheeseman, J. R.; Scalmani, G.; Barone, V.; Mennucci, B.; Petersson, G. A.; Nakatsuji, H.; Caricato, M.; Li, X.; Hratchian, H. P.; Izmaylov, A. F.; Bloino, J.; Zheng, G.; Sonnenberg, J. L.; Hada, M.; Ehara, M.; Toyota, K.; Fukuda, R.; Hasegawa, J.; Ishida, M.; Nakajima, T.; Honda, Y.; Kitao, O.; Nakai, H.; Vreven, T.; Montgomery, J. A., Jr.; Peralta, J. E.; Ogliaro, F.; Bearpark, M.; Heyd, J. J.; Brothers, E.; Kudin, K. N.; Staroverov, V. N.; Kobayashi, R.; Normand, J.; Raghavachari, K.; Rendell, A.; Burant, J. C.; Iyengar, S. S.; Tomasi, J.; Cossi, M.; Rega, N.; Millam, N. J.; Klene, M.; Knox, J. E.; Cross, J. B.; Bakken, V.; Adamo, C.; Jaramillo, J.; Gomperts, R.; Stratmann, R. E.; Yazyev, O.; Austin, A. J.; Cammi, R.; Pomelli, C.; Ochterski, J. W.; Martin, R. L.; Morokuma, K.; Zakrzewski, V. G.; Voth, G. A.; Salvador, P.; Dannenberg, J. J.; Dapprich, S.; Daniels, A. D.; Farkas, Ö.; Foresman, J. B.; Ortiz, J. V.; Cioslowski, J.; Fox, D. J. Gaussian, Inc., Wallingford CT, **2009**.
8. AIMAll (Version 13.10.19), Todd A. Keith, TK Gristmill Software, Overland Park KS, USA, 2013 (aim.tkgristmill.com).
9. S. F. Boys, F. Bernardi, *Mol. Phys.* **2002**, *100*, 65.

4. Z-Matrices

[Cp*Rh(H)$_2$(Bpin)(SiEt$_3$)]

Sum of electronic and zero-point Energies= -1280.690041
Sum of electronic and thermal Energies= -1280.659128
Sum of electronic and thermal Enthalpies= -1280.658183
Sum of electronic and thermal Free Energies= -1280.750068

Lowest frequency vibrations = 31.2, 48.4, 56.2 cm^{-1}

0 1

Rh	-0.32677600	-0.02928000	-0.00777100
Si	2.06102800	-0.17223100	0.02365600
C	2.96674200	1.50560000	-0.11506100
C	4.49382700	1.34096500	-0.08569600
C	2.65215200	-1.01442800	1.64898000
C	2.40595000	-0.16871200	2.90441600
C	2.62518400	-1.27299800	-1.44795700
C	2.46536500	-0.59317300	-2.81380600
C	-1.40351900	-1.77324300	-1.13897500
C	-0.80182500	-2.37042600	0.01675900
C	-1.37276400	-1.75155600	1.17704800
C	-1.13641900	-2.16354600	-2.56197600
C	0.13718100	-3.53913000	0.02719800
C	-1.06435900	-2.13603700	2.59226400
H	0.33719000	0.70329100	-1.23531100
H	0.32578600	0.76355300	1.18924000
H	-1.73433300	-3.03605300	-2.85158500
H	-1.38219400	-1.34842600	-3.24734800
H	-0.35970500	-4.42290400	0.44541100
H	1.03451400	-3.35456800	0.62582600
H	0.45989400	-3.79386700	-0.98532700
H	-0.00487400	-2.38361100	2.71093400
H	-1.29397600	-1.32355400	3.28635100
H	-1.64868600	-3.01279700	2.89788000
H	2.62246100	2.16010700	0.69269200
H	2.64000200	1.99962900	-1.03737900
H	4.82712800	0.88788300	0.85517100
H	4.84420400	0.69879100	-0.90252500
H	5.00325600	2.30520300	-0.18422000
H	3.72676500	-1.21428900	1.52917400
H	2.16914100	-1.99372100	1.75092400
H	1.33312200	-0.00988600	3.06302900
H	2.81034000	-0.64767800	3.80218900
H	2.87392000	0.81826400	2.81383000
H	2.06221700	-2.21315600	-1.41869700
H	3.67874600	-1.53477900	-1.27448400
H	1.41637600	-0.33699900	-3.00244000
H	2.80527700	-1.23853700	-3.63069800
H	3.04372400	0.33630200	-2.86141300
C	-2.40507900	-0.83404900	-0.69049300
C	-2.38664900	-0.82206900	0.73997800
H	-0.08191700	-2.41260500	-2.71548100
C	-3.35847000	-0.06675600	-1.55463400
C	-3.31298000	-0.03429300	1.61496100

H	-4.31657500	-0.59278300	-1.64420300
H	-3.54057800	0.92540300	-1.13472300
H	-2.95704000	0.06788700	-2.56278600
H	-3.48640400	0.95821400	1.19121900
H	-2.89306100	0.09773400	2.61601900
H	-4.27866300	-0.54258700	1.72250600
B	-0.65850700	1.94211300	-0.04291600
O	-1.97867100	2.43774900	-0.05526900
O	0.24315900	3.01876500	-0.05222800
C	-0.48521900	4.27912700	-0.20153300
H	-0.09736700	4.99432700	0.52226400
H	-0.31439000	4.65642800	-1.21178900
C	-1.96103100	3.90092300	0.04455400
H	-2.64210600	4.30950100	-0.70062000
H	-2.30356000	4.17926000	1.04326400

[Cp*Rh(H)$_2$(SiEt$_3$)$_2$]

Sum of electronic and zero-point Energies= -1553.694836
Sum of electronic and thermal Energies= -1553.657794
Sum of electronic and thermal Enthalpies= -1553.656850
Sum of electronic and thermal Free Energies= -1553.761365

Lowest frequency vibrations = 14.5, 37.7, 50.1 cm^{-1}

0 1

Rh	0.05176500	-0.33971000	-0.02081600
Si	-2.03242900	0.83365200	-0.08288100
C	-1.98338000	2.74883100	-0.16832300
C	-3.39504200	3.35614400	-0.15128700
C	-3.02950600	0.26195600	-1.62446900
C	-2.40687200	0.71356300	-2.95149500
C	-3.02966300	0.40856200	1.50791000
C	-2.47376100	1.08927500	2.76603400
C	-0.01393900	-2.33979500	1.24132100
C	-0.98087700	-2.49666000	0.19317200
C	-0.28417700	-2.43911700	-1.05805400
C	-0.29880900	-2.42168900	2.71172500
C	-2.41785900	-2.88805900	0.37230800
C	-0.87244500	-2.69236900	-2.41291000
H	-0.11149900	0.74672300	1.10590500
H	-0.00358300	0.67027900	-1.22239600
H	-0.37375700	-3.46462700	3.04345200
H	0.49557800	-1.94783300	3.29459100
H	-2.58187300	-3.89794600	-0.02247200
H	-3.11466700	-2.21949800	-0.14167900
H	-2.69081900	-2.90141900	1.43055000
H	-1.92406000	-2.39658300	-2.45971500
H	-0.33478200	-2.13888700	-3.18842700
H	-0.81659000	-3.75896800	-2.66773600
H	-1.44981400	3.05686900	-1.07431300
H	-1.39731800	3.12770800	0.67670800
H	-3.98491900	3.01654900	-1.00983800
H	-3.93884300	3.07357100	0.75704600
H	-3.35993300	4.44971500	-0.18827900

H	-4.04559800	0.66775400	-1.51960600
H	-3.13049800	-0.82801100	-1.60882500
H	-1.40354300	0.28915000	-3.07253900
H	-3.01113900	0.40155700	-3.80975600
H	-2.31030300	1.80450700	-2.99279900
H	-3.04492200	-0.67735700	1.64386300
H	-4.07097700	0.71641500	1.33652700
H	-1.43756200	0.78116400	2.95092300
H	-3.06149800	0.83678900	3.65484200
H	-2.48020800	2.18071800	2.66477800
Si	1.84679800	1.24421500	0.09981900
C	1.28909700	-2.25268200	0.63781400
C	1.11953500	-2.31645600	-0.78525200
H	-1.23734400	-1.92053300	2.96787600
C	1.39415500	3.08840400	-0.18783800
C	3.13726900	0.80723200	-1.25164300
C	2.74166900	1.17456900	1.80441600
C	2.59616700	-2.27046000	1.37064900
C	2.19763000	-2.47507700	-1.81690200
C	2.63305400	3.98068700	-0.36306600
H	0.75061500	3.16210900	-1.07150300
H	0.79489000	3.44014400	0.66068100
C	2.61904500	1.04253400	-2.67599400
H	4.02632100	1.42533200	-1.06117300
H	3.45015100	-0.23411000	-1.12049500
C	1.80022500	1.15186000	3.01434400
H	3.41321800	0.30968900	1.82594100
H	3.38746800	2.06297600	1.84579300
H	2.92098300	-3.30050800	1.56344500
H	3.38392100	-1.77525400	0.79531300
H	2.52262900	-1.76040700	2.33578800
H	3.19059800	-2.40745800	-1.36647800
H	2.13717000	-1.72192400	-2.60818000
H	2.12025900	-3.46066500	-2.29209600
H	3.21297900	3.69069200	-1.24586000
H	3.30076000	3.92102400	0.50319200
H	2.35062100	5.03147900	-0.48551800
H	1.70259100	0.46860500	-2.85916500
H	3.35724700	0.75522000	-3.43256700
H	2.37295000	2.09883800	-2.83588200
H	1.19462300	0.23811000	3.01412400
H	2.34952500	1.20042100	3.96052800
H	1.10124800	1.99719500	2.99028300

Compound **1**

Sum of electronic and zero-point Energies= -2031.935061
Sum of electronic and thermal Energies= -2031.881097
Sum of electronic and thermal Enthalpies= -2031.880153
Sum of electronic and thermal Free Energies= -2032.019132

Lowest frequency vibrations = 15.8, 36.3, 41.0 cm^{-1}

0 1

Rh	-0.11535300	1.53974600	-0.24071000
Al	0.05849900	-0.63766400	-1.36289300
Si	0.01262700	0.98842100	2.04422900
H	0.03131900	-0.29056200	-2.95809400
H	-1.19897500	0.42394200	0.06304600
H	1.14916000	0.61244500	0.01337700
N	-1.26577900	-2.07111600	-1.12700000
C	-1.03140800	-3.19051700	-1.83077900
C	0.24114900	-3.53553400	-2.32275600
H	0.28971000	-4.40382300	-2.96497300
C	1.46613100	-3.00544800	-1.88853800
N	1.56115200	-1.87885800	-1.15677400
C	-2.16233000	-4.16034100	-2.08060300
H	-2.36975400	-4.75590500	-1.18501300
H	-1.90149800	-4.84270300	-2.89003100
H	-3.08174800	-3.62727100	-2.33650300
C	2.72696800	-3.75430700	-2.25575600
H	3.16285400	-4.25974100	-1.38940900
H	3.48189000	-3.05819500	-2.63307100
H	2.51468600	-4.50245400	-3.01972400
C	-2.51272800	-1.92742900	-0.42023600
C	-2.77631400	-2.67865700	0.73993400
C	-3.98830800	-2.47415900	1.41011800
H	-4.18456200	-3.04207800	2.31651200
C	-4.93934400	-1.55866100	0.95936100
C	-4.66405100	-0.84562900	-0.21126700
H	-5.39492500	-0.13601300	-0.58984800
C	-3.47013700	-1.01870000	-0.91184800
C	-1.80577300	-3.69846700	1.28796500
H	-2.25863100	-4.69674700	1.29808400
H	-1.53064400	-3.45386900	2.31976100
H	-0.88749700	-3.74677700	0.70043200
C	-6.23563000	-1.35698400	1.70415800
H	-6.63473500	-0.35377600	1.53431000
H	-6.09422700	-1.48773400	2.78043300
H	-6.99573100	-2.07645500	1.37927600
C	-3.20361900	-0.24113500	-2.17136600
H	-2.79852900	-0.87499200	-2.96713700
H	-2.46409400	0.54564000	-1.98400300
H	-4.11722800	0.23694800	-2.53496000
C	2.78626700	-1.61096900	-0.45123500
C	3.63895800	-0.58503000	-0.89284100
C	4.81848200	-0.33579700	-0.18601500
H	5.47599400	0.45851700	-0.52902200
C	5.16752900	-1.07385300	0.94686800

C	4.30895400	-2.09617800	1.35968100
H	4.56272600	-2.68159400	2.23952100
C	3.12165700	-2.38292400	0.67962100
C	3.28091000	0.24145200	-2.09808800
H	2.48214000	0.94898100	-1.84580800
H	2.91231600	-0.37446800	-2.92504300
H	4.14236000	0.81733000	-2.44628900
C	6.41318600	-0.74617500	1.73136700
H	7.18085600	-0.30514300	1.09017200
H	6.83280600	-1.63859600	2.20321600
H	6.19036100	-0.02458600	2.52578400
C	2.22175400	-3.49208700	1.16736300
H	2.39412700	-4.43205300	0.62894200
H	1.17080200	-3.22482200	1.03528700
H	2.39690700	-3.68493400	2.22910300
C	0.20310800	-0.90314000	2.29766400
H	-0.52439500	-1.40671300	1.64652300
H	1.19803300	-1.16726200	1.91878900
C	0.04006000	-1.36922200	3.74953700
H	0.22983700	-2.44474300	3.85660800
H	-0.97402200	-1.17214800	4.11669700
H	0.73798900	-0.84775400	4.41504600
C	1.54777500	1.79099000	2.88556400
H	1.45485600	2.88378900	2.87094200
H	1.52254300	1.49796500	3.94573000
C	2.87346800	1.35826400	2.24470800
H	3.00073600	0.26954700	2.28504500
H	3.73706200	1.81227100	2.74508700
H	2.90634300	1.64071800	1.18644000
C	-1.57262000	1.51351800	2.99318100
H	-1.39417900	1.31234900	4.05967800
H	-1.71677700	2.59560700	2.90093700
C	-2.81327300	0.75481500	2.50486400
H	-2.67098600	-0.33001000	2.58068200
H	-3.01356100	0.96921000	1.44864700
H	-3.71020900	1.01358400	3.07983100
C	-0.46064100	3.86633700	0.07709900
C	-1.51030000	3.36081100	-0.76031000
C	-0.92349200	2.96704700	-2.01816300
C	0.48230400	3.16124100	-1.92731400
C	0.78083800	3.68748900	-0.61864500
C	-0.63249200	4.60987900	1.36772500
H	-1.65142300	4.51085400	1.74969100
H	-0.44177300	5.67974700	1.21696800
H	0.05138300	4.26272600	2.14824000
C	-2.97791100	3.38245500	-0.44613900
H	-3.49280300	2.53166200	-0.90504400
H	-3.45601100	4.30113900	-0.80806400
H	-3.14824800	3.31517600	0.63258600
C	-1.63849800	2.58464200	-3.27932700
H	-1.28467100	1.62435700	-3.67426200
H	-1.46760100	3.34281800	-4.05385600
H	-2.71806400	2.51505800	-3.12308900
C	1.42884200	2.99045500	-3.07694700
H	2.47141800	3.06352700	-2.75679200
H	1.26028300	3.77189900	-3.82909600

H	1.28919800	2.02155500	-3.57070200
C	2.12122300	4.15951100	-0.14021600
H	2.19369100	4.09922900	0.95025100
H	2.30430100	5.20224000	-0.42984100
H	2.93063000	3.55066600	-0.55552800

Compound **2**

Sum of electronic and zero-point Energies= -2330.279966
Sum of electronic and thermal Energies= -2330.219265
Sum of electronic and thermal Enthalpies= -2330.218321
Sum of electronic and thermal Free Energies= -2330.370868

Lowest frequency vibrations = 29.4, 42.4, 48.2 cm^{-1}

0 1

Rh	-1.66387900	-0.12573800	0.75827600
Zn	0.54098000	0.04274600	-0.39883100
Si	-3.27142600	0.04468600	-0.95863800
H	-1.35242400	1.20704100	-0.01817100
H	-1.22902000	-1.07440200	-0.44669000
N	1.61398700	1.64009400	-1.08170200
C	2.45312100	1.48105600	-2.10935300
C	2.80428200	0.23244700	-2.66405700
H	3.42607400	0.28357200	-3.54825800
C	2.64021900	-1.05429100	-2.11982900
N	1.88585800	-1.34016500	-1.04689800
C	3.16665400	2.68092100	-2.70140200
H	3.27850100	2.55172200	-3.78035200
H	4.17162400	2.76061200	-2.27249000
H	2.64085500	3.61546200	-2.50327800
C	3.41321100	-2.17226300	-2.78933500
H	3.96386500	-2.76451700	-2.05278800
H	4.11543900	-1.77679100	-3.52282000
H	2.72287700	-2.85134400	-3.29921700
C	1.52928300	2.89951000	-0.39111700
C	0.40151500	3.72893200	-0.57609900
C	0.32650300	4.92737200	0.14233200
H	-0.53181100	5.57476100	0.00656600
C	1.33291100	5.30443800	1.02757200
H	1.25624200	6.23725800	1.57449200
C	2.43473000	4.47620400	1.20539500
H	3.21823200	4.76610000	1.89885000
C	2.55470200	3.26939100	0.50655100
C	-0.69159400	3.36294400	-1.56835500
H	-0.72360500	2.27110800	-1.63347000
C	-2.07429800	3.84380100	-1.11085600
H	-2.26608000	3.53973600	-0.07625000
H	-2.85615900	3.41084800	-1.74204000
H	-2.16600600	4.93315400	-1.18018300
C	-0.36818600	3.88878600	-2.97815000
H	-0.25328400	4.97855500	-2.96492500
H	-1.17773500	3.63547700	-3.67159000
H	0.55649400	3.44788500	-3.36288700
C	3.77268800	2.39472800	0.77147300
H	3.73957700	1.52808200	0.10037600

C	5.08893000	3.15062300	0.51646000
H	5.22778100	3.94895200	1.25329200
H	5.10675700	3.60849000	-0.47630000
H	5.94016300	2.46696800	0.60004500
C	3.75075700	1.87109000	2.21755100
H	4.63785000	1.26047300	2.41562500
H	2.86012200	1.26217800	2.40673300
H	3.74605900	2.70349500	2.92952600
C	1.98573600	-2.62975800	-0.41182900
C	2.83049100	-2.75483600	0.72026300
C	2.92515400	-3.99927800	1.35157800
H	3.56712200	-4.11314700	2.21622000
C	2.19799100	-5.09770700	0.89776800
H	2.28402300	-6.05296100	1.40289900
C	1.35836400	-4.95739900	-0.19907400
H	0.78286300	-5.80932000	-0.54727200
C	1.23927900	-3.73363400	-0.87191500
C	3.61652400	-1.56071100	1.25389900
H	2.95425700	-0.68460100	1.19288100
C	4.86280600	-1.25161600	0.40050900
H	5.50715300	-2.13644700	0.34385200
H	5.43929400	-0.44055500	0.86130400
H	4.60268800	-0.94103200	-0.61409000
C	4.04458600	-1.73798500	2.71788600
H	4.84343500	-2.48286200	2.80446400
H	3.21236400	-2.05269300	3.35344900
H	4.43486000	-0.79487200	3.10872300
C	0.29415300	-3.65130700	-2.05986400
H	0.38519600	-2.65011800	-2.49736400
C	0.63556900	-4.69379900	-3.14108700
H	1.68923100	-4.65929800	-3.43162300
H	0.02318000	-4.52618500	-4.03271000
H	0.42627200	-5.70630700	-2.77932500
C	-1.16245500	-3.85022700	-1.60511900
H	-1.84292500	-3.77199100	-2.46048400
H	-1.45254400	-3.10460500	-0.85859800
H	-1.29099700	-4.84520700	-1.16340000
C	-2.64919100	0.51124600	-2.72982400
H	-3.47379400	0.25090600	-3.41017000
H	-2.51832900	1.59631400	-2.81521000
C	-1.36968300	-0.20494300	-3.18111300
H	-1.40991200	-1.27843100	-2.95913100
H	-0.48265600	0.20512500	-2.67592400
H	-1.19288100	-0.08918900	-4.25606100
C	-4.12532200	-1.67385400	-1.20418100
H	-3.47073200	-2.26116600	-1.86055700
H	-4.14304700	-2.20116900	-0.24268100
C	-5.53877400	-1.60494700	-1.79977000
H	-5.97465700	-2.60235200	-1.92539700
H	-5.53263100	-1.12373600	-2.78478300
H	-6.21154300	-1.02714400	-1.15611600
C	-4.63775000	1.32678500	-0.48472100
H	-5.31929100	0.84004800	0.22708600
H	-4.15372200	2.14224500	0.06478200
C	-5.43945300	1.89309100	-1.66869400
H	-5.97399700	1.10526100	-2.20768000

H	-4.78477200	2.39315400	-2.39088500
H	-6.18054100	2.62761500	-1.33376200
C	-3.04203400	-0.88068800	2.53882900
C	-2.70150600	0.50060800	2.75187300
C	-1.27734900	0.58290600	2.94684500
C	-0.73728300	-0.73940200	2.84057200
C	-1.82804300	-1.64005000	2.55526500
C	-4.43338000	-1.42971600	2.43768500
H	-5.06614600	-0.82311000	1.78193900
H	-4.91171700	-1.45979800	3.42454100
H	-4.42958700	-2.44900700	2.04323400
C	-3.66835600	1.63108200	2.93809900
H	-3.28904400	2.56053100	2.50138500
H	-3.85489400	1.81705700	4.00363900
H	-4.62944600	1.41331300	2.46555900
C	-0.52452600	1.83662500	3.28568600
H	0.52907300	1.76477600	2.99832000
H	-0.56457700	2.04648800	4.36183500
H	-0.94022400	2.70077400	2.75912800
C	0.67032400	-1.15979200	3.13996500
H	0.98830900	-1.99032200	2.50105400
H	0.76939100	-1.48991900	4.18300700
H	1.37642000	-0.33557600	2.99370200
C	-1.70005900	-3.12788300	2.40847600
H	-2.48644900	-3.53154400	1.76353400
H	-1.76835900	-3.63499900	3.37901700
H	-0.73940400	-3.39913000	1.95677100

Compound **2’**

Sum of electronic and zero-point Energies= -2330.274194
Sum of electronic and thermal Energies= -2330.214099
Sum of electronic and thermal Enthalpies= -2330.213155
Sum of electronic and thermal Free Energies= -2330.363775

Lowest frequency vibrations = 28.0, 34.0, 37.2 cm^{-1}

0 1

Rh	-1.98585600	-0.55284200	0.15530400
Si	-1.41974300	-0.17756000	2.39469000
H	-0.69610500	-1.45737300	0.44352300
H	-1.31073600	0.88363700	0.28067900
N	2.02080500	-1.05068600	-1.13537100
C	2.77708800	-0.56633700	-2.13002300
C	2.68316200	0.73899700	-2.65431900
H	3.32929700	0.93558900	-3.50038000
C	2.05844500	1.87134200	-2.09826600
N	1.15326300	1.82449100	-1.10793300
C	3.87302000	-1.42029000	-2.74062200
H	4.85267600	-1.07577200	-2.39541700
H	3.85220100	-1.31222200	-3.82852700
H	3.76747600	-2.47493400	-2.48603100
C	2.48755000	3.21899200	-2.65013600
H	3.06605300	3.77600500	-1.90730400
H	1.62267200	3.83708600	-2.89770600

H	3.10195100	3.08972300	-3.54153100
C	2.43359200	-2.23671100	-0.42714100
C	3.54253500	-2.15963800	0.44743400
C	3.90991500	-3.29589700	1.17871900
H	4.75867500	-3.23981800	1.85291800
C	3.20593500	-4.48451700	1.05806800
C	2.12168000	-4.55337500	0.18767900
H	1.58719800	-5.48936300	0.10269000
C	1.71567700	-3.44971500	-0.57258400
C	4.36381300	-0.89234100	0.65524900
H	3.98281600	-0.10205100	-0.00225700
C	0.56099200	-3.58770400	-1.56327700
H	-0.14079200	-2.75975500	-1.38273700
C	0.81257700	3.05508700	-0.44057400
C	-0.33512100	3.79669500	-0.81168900
C	-0.53527400	5.04133700	-0.19960800
H	-1.39457000	5.64050400	-0.46861700
C	0.34219100	5.53234200	0.76191200
C	1.41842000	4.75638200	1.17124400
H	2.06961500	5.12320000	1.95528000
C	1.66429100	3.50345500	0.59632500
C	-1.37046400	3.25756100	-1.80246900
H	-1.60163600	2.22976500	-1.48236200
C	2.80698000	2.64537300	1.13951000
H	2.53193800	1.59509200	0.97823800
C	0.46720400	0.17668100	2.60717000
H	1.02333900	-0.63667200	2.11267500
H	0.69565300	1.11031200	2.06855900
C	0.90003700	0.29473500	4.07486400
H	1.97618900	0.47516000	4.17939000
H	0.66321000	-0.62293800	4.62507500
H	0.37870000	1.12260200	4.56904800
C	-2.32788300	1.35658100	3.12095700
H	-3.41015600	1.17313600	3.10918300
H	-2.04371400	1.44170500	4.17969400
C	-2.00596500	2.65949600	2.37267200
H	-0.92612000	2.85790400	2.34351700
H	-2.48637300	3.52648700	2.83944500
H	-2.34619200	2.60238100	1.33181600
C	-1.78871600	-1.72775000	3.47156300
H	-1.66458400	-1.45362300	4.52884800
H	-2.84194800	-2.00302400	3.34178700
C	-0.87847700	-2.91189500	3.11316700
H	0.17873000	-2.65827900	3.25719000
H	-1.00159800	-3.19070500	2.06002400
H	-1.09494300	-3.79317700	3.72676800
C	-4.26743600	-1.12952900	0.45918600
C	-3.62988900	-2.19313100	-0.25506700
C	-3.23604100	-1.68369500	-1.55014300
C	-3.58455000	-0.30970500	-1.60892900
C	-4.18665800	0.05340400	-0.34794000
C	-5.00642100	-1.24079600	1.75884900
H	-4.83001900	-2.20993300	2.23232500
H	-6.08716500	-1.14737300	1.59387600
H	-4.71672300	-0.46283000	2.47310500
C	-3.55570800	-3.62545800	0.18295900

H	-2.70130000	-4.13319800	-0.27243700
H	-4.46105000	-4.17722600	-0.10021200
H	-3.44147100	-3.70579500	1.26828100
C	-2.70364200	-2.47806100	-2.70378400
H	-1.90729500	-1.94496600	-3.23463000
H	-3.49843900	-2.69209800	-3.43025200
H	-2.29960300	-3.43454300	-2.36972600
C	-3.46857500	0.54834400	-2.83210000
H	-3.60177300	1.60340200	-2.58771900
H	-4.23828500	0.28188600	-3.56808600
H	-2.49393000	0.43732800	-3.31967900
C	-4.81113400	1.37178300	-0.00434400
H	-4.72198800	1.58200600	1.06653100
H	-5.87857700	1.38662400	-0.26015200
H	-4.32795600	2.19279100	-0.54112200
C	-2.67445100	4.07044500	-1.76438400
H	-2.51717400	5.07912200	-2.16296200
H	-3.43297700	3.59600100	-2.39273100
H	-3.07284900	4.15793700	-0.74908100
C	-0.88634100	3.19270800	-3.26358200
H	-0.55300400	4.18068700	-3.60192700
H	-0.07313500	2.47727300	-3.39950900
H	-1.71570900	2.88120200	-3.90814100
C	-0.20508100	-4.90534800	-1.38029600
H	-1.06162500	-4.94227000	-2.05785800
H	0.43038700	-5.76364100	-1.62514800
H	-0.56940000	-5.02463000	-0.35534800
C	1.04270400	-3.49467500	-3.02395100
H	1.46650100	-2.51501900	-3.25314200
H	1.80220200	-4.26027300	-3.22094600
H	0.20123800	-3.66612700	-3.70434800
C	5.85159600	-1.11873200	0.32529700
H	6.39092100	-0.16596700	0.34716200
H	6.31411100	-1.78149300	1.06474300
H	5.98798800	-1.57533900	-0.65822000
C	4.23701500	-0.40128300	2.10743000
H	4.86304400	0.48347600	2.26381300
H	3.20189900	-0.14580400	2.35202200
H	4.56982800	-1.17562100	2.80689200
Zn	0.38370600	0.01950600	-0.54707700
C	2.98698100	2.86591000	2.65199400
H	3.45273600	3.83543600	2.85889900
H	2.02669200	2.82775700	3.17628500
H	3.63937200	2.09699300	3.07251100
C	4.15035500	2.87499200	0.41916800
H	4.38089900	3.94555700	0.36613200
H	4.95856000	2.38241600	0.97187600
H	4.14612800	2.46269600	-0.59375000
H	3.49616200	-5.35584200	1.63381700
H	0.16858400	6.50437100	1.20932400

Compound **3**

Sum of electronic and zero-point Energies= -2265.717299
Sum of electronic and thermal Energies= -2265.657090
Sum of electronic and thermal Enthalpies= -2265.656146
Sum of electronic and thermal Free Energies= -2265.806949

Lowest frequency vibrations = 27.0, 29.3, 35.3 cm^{-1}

0 1

Rh	-1.65076100	-0.18453000	0.73724600
Mg	0.56979100	0.00431600	-0.43576900
Si	-3.25943600	-0.36822900	-0.96605100
H	-1.39225900	0.99520000	-0.27847900
H	-1.10563300	-1.29000900	-0.27349800
N	1.51803400	1.64399400	-1.18062400
C	2.36121300	1.52386600	-2.20878200
C	2.82005100	0.28582100	-2.70950700
H	3.43362000	0.35067000	-3.59927100
C	2.76789400	-0.98004400	-2.10088900
N	2.03334400	-1.27196000	-1.01274500
C	2.93965800	2.75034900	-2.88867500
H	2.73787300	2.70039400	-3.96322300
H	4.02623200	2.76954600	-2.76182000
H	2.52477600	3.67789800	-2.49350700
C	3.64706500	-2.05644600	-2.70204300
H	4.29653600	-2.49588300	-1.93836900
H	4.26549900	-1.65451100	-3.50413900
H	3.03318600	-2.86665400	-3.10501200
C	1.30169100	2.90952600	-0.53034200
C	0.11545600	3.63581500	-0.77578400
C	-0.09888900	4.82648300	-0.07219300
H	-1.00249000	5.39636500	-0.25633300
C	0.82719300	5.29653600	0.85494200
H	0.64154800	6.22088700	1.38980200
C	1.99453200	4.57650700	1.08334100
H	2.72155200	4.94887200	1.79840100
C	2.25425000	3.38174500	0.40103200
C	-0.89352000	3.19253800	-1.82434900
H	-0.76431800	2.11606200	-1.97978400
C	-2.33947100	3.43527100	-1.37048600
H	-2.50364300	3.01791300	-0.37184400
H	-3.04166500	2.95531100	-2.05943800
H	-2.58139200	4.50335800	-1.35186000
C	-0.62597700	3.88770400	-3.17158600
H	-0.71447800	4.97462200	-3.06294200
H	-1.35278100	3.55527300	-3.92050900
H	0.37759300	3.66181400	-3.54360700
C	3.56428200	2.65282000	0.67134300
H	3.56981600	1.72023300	0.09443400
C	4.77021300	3.50006700	0.22381000
H	4.85570100	4.40094000	0.84155800
H	4.67348300	3.81717100	-0.81768400
H	5.69796900	2.92774800	0.32926900

C	3.71182200	2.29353800	2.15846800
H	4.66856700	1.79008100	2.33345500
H	2.90679100	1.63136000	2.49332800
H	3.68902500	3.19274900	2.78298600
C	2.24614600	-2.51532400	-0.31446200
C	3.06779800	-2.50254800	0.84239000
C	3.26296700	-3.69968500	1.53905900
H	3.88756300	-3.70987700	2.42342300
C	2.66093600	-4.88543500	1.12302300
H	2.82543800	-5.80234400	1.67733600
C	1.84311400	-4.88139700	0.00116200
H	1.36167800	-5.80111200	-0.31605400
C	1.62123400	-3.70868200	-0.73233500
C	3.73409100	-1.21527200	1.32313900
H	2.99764500	-0.40411900	1.21862200
C	4.95331900	-0.83562000	0.45896500
H	5.66516100	-1.66837800	0.42811500
H	5.46440800	0.03220700	0.89253900
H	4.66786600	-0.57722900	-0.56367800
C	4.16166300	-1.28143500	2.79623700
H	5.01932000	-1.95107100	2.92458900
H	3.35239300	-1.63325500	3.44234600
H	4.46764000	-0.28996200	3.13954300
C	0.68288100	-3.77305400	-1.92740200
H	0.68731700	-2.79184400	-2.41663400
C	1.11516000	-4.83682900	-2.95374900
H	2.15952700	-4.72321800	-3.25706700
H	0.48594900	-4.77454600	-3.84719100
H	1.00013400	-5.84305900	-2.53687400
C	-0.75258200	-4.07593500	-1.46086800
H	-1.43991200	-4.08048300	-2.31461000
H	-1.10105700	-3.33519100	-0.73497400
H	-0.79721400	-5.06366600	-0.98784900
C	-2.66196300	-0.05973200	-2.78014200
H	-3.45098800	-0.45909900	-3.43400700
H	-2.62359300	1.01899700	-2.97696500
C	-1.31924400	-0.70192300	-3.15218200
H	-1.24968700	-1.73262400	-2.78647700
H	-0.47366500	-0.13771600	-2.72993000
H	-1.15836000	-0.71747700	-4.23566900
C	-3.96902300	-2.16564600	-0.95545300
H	-3.23528200	-2.80717700	-1.45944300
H	-4.00291000	-2.50443000	0.08800600
C	-5.34787700	-2.32312600	-1.61221000
H	-5.68885900	-3.36440400	-1.59169800
H	-5.32950900	-2.00557500	-2.66122600
H	-6.10330000	-1.71829800	-1.09826500
C	-4.74648800	0.83559700	-0.66921100
H	-5.40421300	0.37176700	0.07948400
H	-4.36385900	1.75510800	-0.21263400
C	-5.55532200	1.17760200	-1.93171300
H	-5.95002100	0.27806100	-2.41381200
H	-4.93648700	1.69715000	-2.67127600
H	-6.40480400	1.82951600	-1.69855400
C	-2.97693500	-0.68515500	2.65620700
C	-2.68351200	0.72183800	2.63370800

C	-1.25865600	0.88406100	2.78368900
C	-0.67137100	-0.41867100	2.86754100
C	-1.73662000	-1.38969800	2.75510300
C	-4.34713300	-1.29471400	2.66752800
H	-5.00814600	-0.83702500	1.92435400
H	-4.81950100	-1.17343000	3.65017300
H	-4.30392100	-2.36590400	2.45376500
C	-3.68966100	1.83278900	2.65711600
H	-3.34638900	2.69735400	2.07999200
H	-3.87767400	2.17430900	3.68330700
H	-4.64461800	1.51009800	2.23317600
C	-0.55132900	2.20376800	2.89799600
H	0.51709700	2.11126500	2.67747800
H	-0.64779000	2.62056800	3.90831400
H	-0.95632900	2.93694700	2.19285300
C	0.75657500	-0.75068200	3.18748000
H	1.11347700	-1.61490000	2.61535200
H	0.87884600	-0.99019800	4.25232800
H	1.42125800	0.09236700	2.97042800
C	-1.55334400	-2.87609900	2.84880700
H	-2.34934700	-3.40813500	2.32015600
H	-1.55739900	-3.21597900	3.89204400
H	-0.60348200	-3.18493100	2.40035100

Compound **3’**

Sum of electronic and zero-point Energies= -2265.713233
Sum of electronic and thermal Energies= -2265.652743
Sum of electronic and thermal Enthalpies= -2265.651799
Sum of electronic and thermal Free Energies= -2265.806042

Lowest frequency vibrations = 8.9, 26.1, 29.6 cm^{-1}

0 1

Rh	2.08288500	-0.14733100	0.11599000
Si	1.27054700	-0.15695100	2.30174500
H	1.11537300	1.12217800	0.20428300
H	0.99314500	-1.32044200	0.11252900
N	-1.53274500	1.62559000	-1.19713100
C	-2.43983600	1.47497100	-2.17742200
C	-2.86820800	0.22842500	-2.66923500
H	-3.54966500	0.27138200	-3.51026200
C	-2.69393400	-1.05093000	-2.10212600
N	-1.83946100	-1.32298100	-1.10413900
C	-3.05646000	2.69093700	-2.84967100
H	-4.10022400	2.50472100	-3.10859800
H	-2.51645200	2.89710300	-3.78033900
H	-2.99594800	3.58291100	-2.22499300
C	-3.59525000	-2.13771100	-2.66596700
H	-4.58832900	-2.08317700	-2.21043800
H	-3.20139100	-3.13699600	-2.48362300
H	-3.71442300	-1.99386900	-3.74253700
C	-1.47270600	2.86386700	-0.46220500
C	-2.47757100	3.09085100	0.51004400
C	-2.45835800	4.28462200	1.23944700

H	-3.23044000	4.47032900	1.97841400
C	-1.45951800	5.22758000	1.03501600
C	-0.45421400	4.97655600	0.10502900
H	0.32500800	5.71528100	-0.02544000
C	-0.42949600	3.80021200	-0.65658200
C	-3.57517400	2.07040400	0.78996700
H	-3.24411100	1.10587100	0.38667800
C	0.70303600	3.55239400	-1.65288900
H	1.13304800	2.56849000	-1.41016500
C	-1.88613400	-2.61827700	-0.47420800
C	-1.02801100	-3.64855900	-0.92663100
C	-1.14307000	-4.91206200	-0.33311000
H	-0.50296500	-5.72071100	-0.66040500
C	-2.06287800	-5.15287100	0.68128800
C	-2.85868300	-4.11599100	1.15323200
H	-3.54221900	-4.30911400	1.97012400
C	-2.78006300	-2.83007200	0.60299500
C	0.00643700	-3.41254400	-2.02803700
H	0.47499800	-2.43718200	-1.83206000
C	-3.61172400	-1.69693500	1.20980600
H	-2.97754000	-0.80246700	1.21900700
C	-0.66253600	-0.13796500	2.37743900
H	-1.05571700	0.73891400	1.82905000
H	-1.02177700	-1.04857500	1.87325400
C	-1.19391200	-0.10743800	3.81859500
H	-2.28760000	-0.13778300	3.85911800
H	-0.86675100	0.80221000	4.33487400
H	-0.81942200	-0.96653000	4.38650300
C	1.75563900	-1.76097100	3.25078000
H	2.84734700	-1.86272700	3.25633600
H	1.45466700	-1.64210400	4.30121400
C	1.11675500	-3.02108900	2.64599700
H	0.02076100	-2.96470500	2.65287000
H	1.40212200	-3.92365700	3.19734200
H	1.42481200	-3.14927300	1.60172500
C	1.83102400	1.40776800	3.26905500
H	1.51745900	1.29430000	4.31636700
H	2.92703100	1.44839900	3.27613700
C	1.25496100	2.70189300	2.67179100
H	0.15812300	2.67430500	2.61612000
H	1.61938700	2.85285100	1.64878100
H	1.53078000	3.58183700	3.26314600
C	4.40616800	-0.14062900	0.65391600
C	4.12912500	0.96376600	-0.21289900
C	3.75313400	0.43620900	-1.50698300
C	3.73565200	-0.97722500	-1.41072200
C	4.09592000	-1.34334000	-0.05678500
C	5.01750900	-0.05318500	2.01995600
H	5.00995600	0.97669300	2.38677300
H	6.06276800	-0.38555800	1.99553400
H	4.49426200	-0.67195400	2.75564700
C	4.38467500	2.40648600	0.10332700
H	3.83612000	3.05653200	-0.58189600
H	5.45044600	2.65249300	0.01311700
H	4.06687500	2.65753800	1.12040400
C	3.53193600	1.22704600	-2.76148800

H	2.87034400	0.69434500	-3.45200400
H	4.47794600	1.41543900	-3.28567000
H	3.07582100	2.19782900	-2.55006200
C	3.52396300	-1.92885900	-2.55031600
H	3.54680500	-2.96096300	-2.19548400
H	4.31928600	-1.81966800	-3.29796300
H	2.56750100	-1.77117100	-3.06151900
C	4.31133200	-2.73740800	0.45239200
H	4.18104800	-2.78325400	1.53818800
H	5.32252400	-3.09772600	0.22265000
H	3.59726700	-3.43821600	0.01003400
C	1.12233100	-4.46649300	-2.01052400
H	0.74102800	-5.45496400	-2.28839000
H	1.88679000	-4.20104600	-2.74350900
H	1.59207600	-4.53849400	-1.02397800
C	-0.60372200	-3.36595000	-3.44268700
H	-1.19976800	-4.26603300	-3.63276100
H	-1.23657200	-2.48972200	-3.59325500
H	0.19895900	-3.32806600	-4.18734100
C	1.82464600	4.59457900	-1.53696700
H	2.65563700	4.32136100	-2.19455300
H	1.47338300	5.58396000	-1.85179900
H	2.20833100	4.67203300	-0.51540500
C	0.22407900	3.50652400	-3.11610400
H	-0.39292700	2.62854000	-3.30935600
H	-0.35242900	4.40634300	-3.35950800
H	1.08843800	3.46345100	-3.78809700
C	-4.89975500	2.43923600	0.09689600
H	-5.68282700	1.72620300	0.37651400
H	-5.22135500	3.44036100	0.40611100
H	-4.80696400	2.42936200	-0.99064000
C	-3.82161800	1.89842800	2.29719500
H	-4.44874400	1.01742800	2.47302500
H	-2.88338300	1.77562600	2.84523400
H	-4.34942100	2.76236000	2.71560500
Mg	-0.42726700	0.03746700	-0.56667600
H	-1.45305100	6.14996500	1.60441000
H	-2.14280200	-6.14141900	1.11899800
C	-4.00441800	-1.99435400	2.66637700
H	-4.41617100	-1.09323100	3.13029000
H	-4.77679600	-2.76945900	2.72076400
H	-3.14221300	-2.32137100	3.25608100
C	-4.87806000	-1.33876100	0.40755300
H	-4.63970700	-0.78942500	-0.50712600
H	-5.44348900	-2.24126400	0.14925800
H	-5.52699500	-0.69611900	1.01446500

Compound **4**

Sum of electronic and zero-point Energies= -1504.569458
Sum of electronic and thermal Energies= -1504.525970
Sum of electronic and thermal Enthalpies= -1504.525026
Sum of electronic and thermal Free Energies= -1504.647675

Lowest frequency vibrations = 8.4, 18.2, 40.0 cm^{-1}

0 1

Rh	-0.00711700	1.44833200	-0.27644700
H	-0.16643700	0.61625100	-1.65715100
H	-1.54809300	1.15913000	-0.09994900
C	1.17359400	3.42711600	-0.99001500
C	2.07497000	2.60849700	-0.21244600
C	1.57359800	2.55090100	1.12391800
C	0.32704600	3.26275700	1.15588000
C	0.10980800	3.84510000	-0.14815000
C	1.38000800	3.80766800	-2.42579400
H	0.42855100	4.01683900	-2.92249300
H	1.87083100	3.00156500	-2.97896400
H	2.00861100	4.70302700	-2.51662200
C	3.38271600	2.05167700	-0.69165000
H	3.30575200	1.67129100	-1.71583600
H	3.70535300	1.21567000	-0.06276400
H	4.17772900	2.81032900	-0.67827800
C	2.26751100	1.93540200	2.30280900
H	2.87671100	1.07705600	2.00111000
H	1.54900800	1.59000300	3.05287600
H	2.93375700	2.65783500	2.79323300
C	-0.49939800	3.54128700	2.37634300
H	-0.39277100	2.74285400	3.11685900
H	-1.56150800	3.61526400	2.12506900
H	-0.20352700	4.48322300	2.85717400
C	-1.01831300	4.76327400	-0.51331600
H	-1.93920400	4.48452400	0.00683300
H	-1.22603400	4.72891500	-1.58657900
H	-0.79060100	5.80511300	-0.25193500
Al	-0.29822300	-0.75939000	-0.05189200
N	0.91982500	-2.24066500	-0.01722700
C	0.58949800	-3.54363500	0.02772000
C	-0.73465900	-3.99707000	0.04261400
H	-0.88110800	-5.06706200	0.08012700
C	-1.89032400	-3.20487000	0.02447600
N	-1.86801000	-1.86116000	-0.02254300
C	1.69413400	-4.57103100	0.07246000
H	2.34026500	-4.40233200	0.93980100
H	2.33232000	-4.49178100	-0.81336400
H	1.28331600	-5.57870300	0.12485100
C	-3.22683800	-3.90448000	0.07142300
H	-3.83276900	-3.64019500	-0.80070000
H	-3.79303100	-3.58826700	0.95347000
H	-3.09658900	-4.98561600	0.10091800
C	2.31792600	-1.86868900	-0.01234500
C	3.00370000	-1.73901300	-1.22947700

C	4.35744300	-1.39595800	-1.19469500
H	4.89699200	-1.29285300	-2.13204900
C	5.02508400	-1.15913600	0.01050500
C	4.30633500	-1.27211400	1.20404500
H	4.80508500	-1.07504800	2.14909700
C	2.95158600	-1.61821300	1.21353600
C	2.28200800	-1.93476600	-2.53782400
H	1.52619500	-1.15142000	-2.67345800
H	1.76390400	-2.89894800	-2.58344600
H	2.97939400	-1.88108100	-3.37629500
C	6.46961700	-0.72558300	0.01565800
H	7.02521600	-1.18542200	-0.80589000
H	6.96232900	-0.99211900	0.95404700
H	6.54376200	0.36175100	-0.10179800
C	2.17335200	-1.69719900	2.50215400
H	1.71576000	-2.68258400	2.64561000
H	1.36208300	-0.95684600	2.50449900
H	2.81859800	-1.48872500	3.35830300
C	-3.12410800	-1.14113200	-0.02463100
C	-3.66710000	-0.71435100	1.19516500
C	-4.86954400	-0.00387100	1.17414700
H	-5.29526300	0.33751500	2.11351200
C	-5.52373500	0.29319800	-0.02464400
C	-4.94024600	-0.12436800	-1.22424400
H	-5.42229400	0.12242200	-2.16592200
C	-3.73869200	-0.83626100	-1.24681300
C	-2.93382700	-0.96886800	2.48669800
H	-2.66777800	-2.02447100	2.60977900
H	-3.53862300	-0.66466000	3.34345300
H	-2.00087200	-0.38941900	2.50977400
C	-6.83446200	1.04052800	-0.02411400
H	-6.91129300	1.70056700	0.84359000
H	-7.68300900	0.34793800	0.00935200
H	-6.94051300	1.65001100	-0.92522500
C	-3.08520300	-1.22377400	-2.54804600
H	-2.83597500	-2.28998600	-2.58385300
H	-2.15146900	-0.66368900	-2.68347700
H	-3.73923000	-0.99612300	-3.39230300
